# A Combined Colon Organoid‐Sensory Neuron Model Reveals Epithelial Contribution to Moringin Efficacy Against Painful Inflammatory Bowel Disease

**DOI:** 10.1002/ptr.70303

**Published:** 2026-03-23

**Authors:** Francesco Margiotta, Elena Lucarini, Alessandra Toti, Maria Giovanna Cataldi, Clara Ciampi, Gina Rosalinda De Nicola, Lorenzo Di Cesare Mannelli, Carla Ghelardini

**Affiliations:** ^1^ Department of Neuroscience, Psychology, Drug Research and Child Health—NEUROFARBA—Pharmacology and Toxicology Section University of Florence Florence Italy; ^2^ European Biomedical Research Institute of Salerno (EBRIS) Salerno Italy; ^3^ Research Centre for Vegetable and Ornamental Crops, Council for Agricultural Research and Economics (CREA) Pescia Italy

**Keywords:** epithelial‐neuronal signaling, IBD, *Moringa oleifera*, organoids, visceral pain

## Abstract

Visceral pain is a major symptom of inflammatory bowel diseases (IBDs), requiring effective treatment strategies. Gut epithelium, beyond maintaining barrier integrity and microbiota homeostasis, modulates neurosensorial circuitries, influencing visceral sensitivity. *Moringa oleifera* constituents show beneficial properties that may counteract inflammation‐induced epithelial dysfunction and visceral hypersensitivity. Among these, we investigated moringin (MOR), the isothiocyanate derived from the myrosinase (MYR)‐mediated hydrolysis of glucomoringin (GMG). A three‐stage experimental strategy was applied. First, murine colon organoids were exposed to a pro‐inflammatory cytokine cocktail (CKs; TNF‐α, IL‐1β, IL‐6; 10 ng mL^−1^, 6 h) to model epithelial inflammatory stress and treated with MOR (2–30 μM). Second, epithelial–neuronal communication was assessed by exposing primary dorsal root ganglion (DRG) neurons to conditioned media from inflamed organoids, with (CM^CKs + MOR 30 μM^) or without (CM^CKs^) MOR treatment. Third, the therapeutic relevance of these findings was validated in vivo using a dextran sulfate sodium (DSS)–induced colitis model, in which mice received oral administration of MYR‐bioactivated GMG (GMG + MYR) at different doses (30–100 mg kg^−1^), followed by behavioral and histological assessments. CKs‐treated organoids showed cytotoxicity, increased superoxide dismutase activity, and upregulation of *Ccl2*, *Ccnd1*, *Mki67,* and *Pyy*. MOR 30 μM co‐treatment normalized oxidative stress and gene expression, although cytotoxicity remained unaffected. CM^CKs^ increased c‐Fos and CGRP expression in DRG neurons, while CM^CKs + MOR 30 μM^ prevented this response, indicating MOR modulation of epithelial inflammation‐driven neuronal activation. In DSS‐treated mice, oral GMG (30–100 mg kg^−1^) + MYR dose‐dependently reduced visceral hypersensitivity, pain‐associated behavioral alterations, and colonic damage. Overall, MOR/GMG + MYR showed gut‐protective and analgesic effects under intestinal inflammation. Intestinal organoids effectively model inflammation and therapeutic responses, potentially reducing animal use in IBD research.

## Graphical abstract

Experimental workflow and main findings of the study.

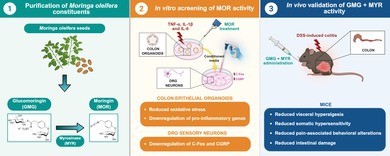

**Abbreviations**
ATP: adenosine triphosphate
AWR: abdominal withdrawal reflex
CCL2: chemokine (C‐C motif) ligand 2
CGRP: calcitonin gene‐related peptide
CK: cytokine
CM: conditioned media
CRD: colorectal distension
DAI: disease activity index
DAPI: 4′,6‐diamidino‐2‐phenylindole
DMEM: Dulbecco's modified Eagle medium
DRG: dorsal root ganglion
DSS: dextran sulfate sodium
EDTA: ethylenediaminetetraacetic acid
EGF: epidermal growth factor
FBS: fetal bovine serum
GMG: glucomoringin
H&E: hematoxylin and eosin
HEPES: 4‐(2‐hydroxyethyl)‐1‐piperazineethanesulfonic acid
HPLC: high performance liquid chromatography
IBD: inflammatory bowel disease
IL: interleukin
KEAP1: Kelch‐like ECH‐associated protein 1
LDH: lactate dehydrogenase
MLKL: mixed lineage kinase domain‐like pseudokinase
MOR: moringin
mTOR: mammalian target of rapamycin
MYR: myrosinase
NADPH: nicotinamide adenine dinucleotide phosphate
NF‐κB: nuclear factor‐kappa B
NGF: nerve growth factor
NRF2: nuclear factor erythroid 2‐related factor 2
PBS: phosphate‐buffered saline
PCR: polymerase chain reaction
PI3K: phosphatidylinositol‐3‐kinase
PYY: peptide YY
RIPA: radioimmunoprecipitation assay
RIPK: receptor interacting protein kinase
ROI: region of interest
ROS: reactive oxygen species
SOD: superoxide dismutase
TNF: tumor necrosis factor
TRP: transient receptor potential

## 1 Introduction

Chronic abdominal pain affects a large proportion of patients with inflammatory bowel disease (IBD) and remains a major unmet therapeutic need due to the absence of effective and safe analgesic options (Hashash et al. 2024; Keefer et al. 2024). At present, conventional and biological therapies can dampen gut inflammation, but sedation of pain symptoms, particularly those deriving from visceral sensitivity, is inconsistent and often accompanied by unacceptable side effects (Klemm and Moosavi 2024; van Gils et al. 2025). At the intestinal level, inflammation‐driven disruption of epithelial barrier integrity, dysbiosis, immune activation, and altered neurosensory signaling converge to drive visceral hypersensitivity in IBD (Barbara et al. 2021; Ford et al. 2024). Importantly, the intestinal epithelium is now recognized as an active regulator of visceral nociception rather than a passive barrier. Beyond its barrier and mucus/microbiome‐interface functions, epithelial cells directly communicate with sensory nerve fibers through the release of cytokines (CKs), reactive oxygen species (ROS), adenosine triphosphate (ATP), lipid mediators, and neurotrophic factors, thereby shaping sensory neuron excitability in both healthy and inflammatory conditions (Najjar et al. 2021; Bayrer et al. 2023; Ohara and Hsiao 2025). In inflamed states, oxidative stress‐related epithelial signaling, in particular, represents a key driver of neuronal hyperexcitability in chronic visceral pain states (Lapointe and Altier 2011). Recent in vitro evidence has further demonstrated that the epithelium‐to‐neuron signaling regulating the excitability of dorsal root ganglion (DRG) nociceptors is affected by post‐inflammatory epithelial dysfunction and dysbiosis, even in the absence of overt immune activation (Margiotta et al. 2025). These findings highlight epithelial stress responses as critical upstream drivers of neuronal hypersensitivity and identify the epithelium–neuron axis as a key, yet underexplored, therapeutic target for chronic visceral pain in IBD.

Phytochemicals derived from medicinal plants offer a promising strategy to modulate this complex axis due to their pleiotropic actions on epithelial integrity, oxidative stress and inflammatory signaling. *Moringa oleifera* is a rich source of 4‐(α‐l‐rhamnosyloxy)‐benzyl glucosinolate (glucomoringin, GMG), the precursor of the bioactive 4‐(α‐Lrhamnosyloxy)‐benzyl isothiocyanate (moringin, MOR) released upon hydrolysis catalyzed by the enzyme myrosinase (𝛽‐thioglucoside glucohydrolase; EC 3.2.1.147, MYR) at neutral pH (Fahey, Wade, Stephenson, Shi, et al. 2019). Isothiocyanates are well‐characterized antioxidant, anti‐inflammatory and neuroprotective agents (Rajan et al. 2016; Jaja‐Chimedza et al. 2017; Kim et al. 2017; Lucarini, Micheli, Di Cesare Mannelli, et al. 2022; Razis et al. 2025). In mice with dextran sulfate sodium (DSS)‐induced ulcerative colitis, oral MOR significantly improved body weight loss and histological colon damage, upregulated tight junction and mucin proteins, and modulated pathways critically involved in epithelial stress responses during intestinal inflammation, including NRF2/NF‐κB and PI3K/AKT/mTOR (Zhang, Zhao, et al. 2024). In addition to epithelial‐protective effects, isothiocyanates from *M. oleifera* have also been reported to modulate transient receptor potential (TRP) channels, including TRPA1 and TRPV1, which play a central role in visceral nociception and sensory neuron sensitization (Borgonovo et al. 2020). Furthermore, *M. oleifera* extracts exhibit analgesic effects in different inflammatory pain models (Adedapo et al. 2015; Martínez‐González et al. 2017; Palomino‐Pacheco et al. 2024). However, despite these converging lines of evidence, it remains unknown whether MOR can attenuate visceral hypersensitivity by acting on the inflamed intestinal epithelium and, consequently, interrupting epithelial‐derived pro‐nociceptive signaling to sensory neurons.

Based on this unaddressed mechanistic gap, we hypothesized that MOR/GMG + MYR could reduce colitis‐associated visceral pain by preserving epithelial homeostasis and dampening epithelium‐to‐neuron sensitizing signals. To this aim, we implemented a three‐stage experimental strategy. First, mouse colonic crypts were isolated and cultured into 3D epithelial organoids. A well‐defined CK cocktail of TNF‐α, IL‐1β, and IL‐6 (10 ng mL^‐1^) was then applied to induce controlled epithelial stress, mimicking key aspects of inflammation‐driven barrier dysfunction and inflammatory gene induction frequently observed in IBD epithelium (d'Aldebert et al. 2020; Di Giorgio et al. 2023; Flood et al. 2024). Organoids were concurrently co‐treated with MOR (2–30 μM) to evaluate whether this phytochemical can modulate epithelial stress responses, particularly oxidative and inflammatory pathways, in a human‐relevant reductionist system. Secondarily, the combination of colon organoids and primary DRG neurons was used to evaluate the efficacy of MOR in counteracting neuronal activation associated with epithelial inflammation. Finally, to validate the in vitro observations in an intact physiological context, we administered GMG + MYR orally (30–100 mg kg^−1^ + MYR) across escalating doses in mice undergoing DSS‐induced colitis, a commonly used model of IBD featuring visceral pain and intestinal barrier damage (Chassaing et al. 2014). Visceral sensitivity was quantified using the abdominal withdrawal reflex (AWR) to colorectal distension (CRD), while colonic tissue damage was assessed histologically. This in vivo arm served to test the functional and therapeutic relevance of the organoid screening results. The overall aim of this combinatorial approach is twofold: (1) to support colon epithelial organoids as a reductionist platform for preliminary efficacy screening of epithelium‐targeting treatments in IBD, thereby reducing reliance on animal models and (2) to establish a mechanistically informed pipeline linking in vitro organoid discoveries with in vivo functional outcomes, aimed at developing multitarget phytotherapeutics capable of protecting epithelial integrity and reducing visceral hypersensitivity in chronic intestinal inflammation.

## 2 Materials and Methods

### 2.1 Glucomoringin, Moringin, and Myrosinase Purification

#### 2.1.1 Glucomoringin Purification

GMG was isolated from *M. oleifera* seeds (cake powder PKM‐2 provided by Indena India Pvt. Ltd.; Bangalore, India) and purified in two sequential steps, by anion exchange and size exclusion chromatography, according to a well‐established procedure available at our labs and already reported extensively in a previous study (Galuppo, Giacoppo, Iori, De Nicola, Bramanti et al. 2015). The purity was assessed by high performance liquid chromatography (HPLC) analysis of the desulfo‐derivative according to the EU standard procedure (ISO 9167:2019; ISO 2019), yielding about 99% based on peak area value and more than 95% on weight basis due to its marked hygroscopic properties.

#### 2.1.2 Myrosinase Purification

MYR was purified as already reported (Galuppo, Giacoppo, Iori, De Nicola, Bramanti et al. 2015) and stored at 4°C in sterile saline solution at neutral pH until use. One MYR unit was defined as the amount of enzyme able to hydrolyze 1 μmol min^−1^ of sinigrin (prop‐2‐enyl glucosinolate) at neutral pH and 37°C.

#### 2.1.3 Moringin Isolation

GMG was hydrolyzed with MYR in 0.1 M phosphate buffer pH 6.5 at 37°C. After monitoring the quantitative conversion of pure GMG into MOR by HPLC, acetonitrile was added to the mixture up to a final concentration of 20%, and afterward MOR was purified by reverse‐phase chromatography. Fractions containing MOR were pooled together and freeze‐dried yielding pure MOR (Razis et al. 2025).

#### 2.1.4 Glucomoringin Bioactivation

Fresh GMG + MYR solutions were prepared for the in vivo study. GMG was bioactivated with MYR enzyme immediately before administration in mice. Purified GMG powder was dissolved in PBS solution pH 7.2 and hydrolyzed with MYR (23.4 U mL^−1^) at 37°C for 30 min (100 mg kg^−1^ GMG + 250 μL kg^−1^ MYR; 10 mL kg^−1^), yielding MOR quantitatively, as previously reported (Galuppo, Giacoppo, Iori, De Nicola, Bramanti et al. 2015).

### 2.2 Animals

Male C57BL6/N mice (6–8 weeks old), weighing 25–30 g, were purchased from Charles River Laboratories (Lecco, Italy). Animals were housed in groups of four in cages (26 × 41 cm) at Ce.S.A.L. (Centro Stabulazione Animali da Laboratorio, University of Florence) under controlled environmental conditions (23°C ± 1°C; 12‐h light/dark cycle, lights on at 07:00). Mice had ad libitum access to tap water and a standard laboratory diet (Teklad Global Diet #2018; 18.5% protein, 5.5% fat; Inotiv, distributed by Mucedola, Milan, Italy). All experimental procedures were conducted in accordance with Guidance on the operation of the Animals (Scientific Procedures) Act 1986 and Directive 2010/63/EU of the European Parliament and Council on the protection of animals used for scientific purposes, and followed the guidelines of the International Association for the Study of Pain (IASP). The ethical standards of the University of Florence comply with the Guide for the Care and Use of Laboratory Animals (NIH Publication No. 85‐23, revised 1996; Assurance No. A5278‐01). The study was approved by the Italian Ministry of Health (No. 17E9C.N.B5Z and 1046/2023‐PR) and by the Animal Subjects Review Board of the University of Florence. All efforts were made to minimize animal suffering and to reduce the number of animals used, in accordance with the ARRIVE 2.0 guidelines (du Sert et al. 2020).

### 2.3 Murine Colon Organoid Culture Protocol

Murine colonic organoids were generated based on the protocol described by Fan et al. (Fan et al. 2019), with minor modifications. Briefly, the colon was excised from male C57BL6/N mice (6–8 weeks old) and thoroughly rinsed in ice‐cold PBS supplemented with 100 U mL^−1^ penicillin and 100 μg mL^−1^ streptomycin (Merck, Milan, Italy). To isolate intestinal crypts, tissues were incubated in a non‐enzymatic dissociation solution containing 20 mM EDTA in PBS for 35 min at 37°C, followed by vigorous vortexing to obtain four crypt‐containing fractions. Crypts were then embedded in Matrigel Growth Factor Reduced Basement Membrane Matrix (Corning, #356231, Tewksbury, MA, USA), supplemented with 50 ng mL^−1^ recombinant murine EGF, 500 ng mL^−1^ recombinant human R‐Spondin‐1, 100 ng mL^−1^ recombinant murine Noggin, 100 ng mL^−1^ recombinant murine Wnt‐3a (all from Peprotech‐Life Technologies, Milan, Italy), N‐2 and B‐27 serum‐free supplements (Gibco‐Life Technologies, Milan, Italy), and 1 μM *N*‐acetyl‐l‐cysteine (Merck, Milan, Italy). Approximately 1000 crypts per well were plated in 50 μL Matrigel domes into 24‐well culture plates (Corning, Tewksbury, MA, USA). After allowing the Matrigel to polymerize for 30 min at 37°C, wells were overlaid with organoid culture medium consisting of Advanced DMEM/F‐12 (Gibco‐Life Technologies, Milan, Italy) supplemented with 100 U mL^−1^ penicillin, 100 μg mL^−1^ streptomycin, 2 mM GlutaMax supplement (Gibco‐Life Technologies, Milan, Italy), 10 μM HEPES, and the same combination of growth factors and supplements as listed above. Medium was refreshed every 2–3 days.

For routine passaging, Matrigel domes were disrupted using ice‐cold PBS, and organoids were harvested by centrifugation at 200*g* for 5 min at 4°C. This washing step was repeated twice. To remove residual Matrigel, organoids were incubated with Cell Recovery Solution (Corning, Tewksbury, MA, USA) for 30 min at 4°C, followed by two additional centrifugation steps at 100*g* for 5 min in ice‐cold PBS. Organoids were replated at a density of approximately 750 per 50 μL Matrigel dome per well in 24‐well plates. Cultures were maintained in a humidified incubator at 37°C with 5% CO_2_. All experimental procedures involving organoids were carried out 7 days after a passage step to ensure consistency.

### 2.4 Primary Murine Dorsal Root Ganglion (DRG) Neuron Culture Protocol

Primary DRG neurons were isolated from male C57BL6/N mice (6–8 weeks old) following the protocol by Perner and Sokol (2021), with slight modifications. In brief, the spinal column was excised and bisected longitudinally. DRGs were carefully dissected and collected in ice‐cold DMEM, high glucose (Merck, Milan, Italy) supplemented with 10% Fetal Bovine Serum (FBS; Euroclone, Milan, Italy), 100 U mL^−1^ penicillin, 100 μg mL^−1^ streptomycin, and 2 mM L‐glutamine (all from Merck, Milan, Italy). The ganglia were enzymatically dissociated by incubation in PBS containing 1.25 mg mL^−1^ Collagenase A and 2.5 mg mL^−1^ Dispase II (both from Merck, Milan, Italy) for 30 min at 37°C. Following digestion, mechanical dissociation was performed by triturating the tissue 10–20 times sequentially through needles of increasing gauge (18G, 23G, and 26G). Approximately 2.5 × 10^3^ DRG neurons were seeded per well onto 13 mm glass coverslips pre‐coated with 30 μL of laminin (10 μg mL^−1^; Merck, Milan, Italy), placed in 24‐well culture plates. Cells were maintained in Neurobasal‐A medium (Gibco‐Life Technologies, Milan, Italy) supplemented with 100 U mL^−1^ penicillin, 100 μg mL^−1^ streptomycin, 2 mM GlutaMax supplement, B‐27 serum‐free supplement (Gibco‐Life Technologies, Milan, Italy), and 50 ng mL^−1^ recombinant murine β‐NGF (Invitrogen‐Life Technologies, Milan, Italy).

### 2.5 Colon Organoid Treatments

Colon organoids were treated 7 days after the passage for 3, 6, or 24 h with a mix of CKs consisting of 10 ng mL^−1^ recombinant murine TNF‐α, 10 ng mL^−1^ recombinant murine IL‐1β and 10 ng mL^−1^ recombinant murine IL‐6 (all from Peprotech‐Thermo Fisher Scientific, Italy) in organoid medium.

MOR was dissolved in dimethyl sulfoxide. Colon organoids were treated 7 days after the passage for 6 h with MOR 2, 10, or 30 μM in organoid medium. Control organoids received an equal amount of vehicle.

### 2.6 Preparation of Conditioned Media From Organoids and DRG Neuron Treatments

After 6 h of treatment with CKs and/or MOR, the organoid medium was replaced with fresh organoid medium. After 18 h of conditioning, conditioned media (CM) from nontreated organoids (CM^CTR^), CKs‐treated organoids (CM^CKs^), and CKs + 30 μM MOR‐treated organoids (CM^CKs + MOR 30 μM^) were collected, centrifuged at 200*g* for 5 min, and transferred into new tubes. CM were immediately used to treat DRG neurons for 48 h, 24 h after their isolation.

### 2.7 Protein Extraction From Murine Colon Organoids

Matrigel domes were disrupted using ice‐cold PBS, and organoids were harvested by centrifugation at 200*g* for 5 min at 4°C. This washing step was repeated twice. To remove residual Matrigel, organoids were incubated with Cell Recovery Solution for 30 min at 4°C, followed by two additional centrifugation steps at 200*g* for 5 min in ice‐cold PBS. Following the final centrifugation, supernatants were carefully removed, and organoid pellets were lysed in radioimmunoprecipitation assay buffer. Subsequently, lysates were clarified by centrifugation at 13,000*g* for 15 min at 4°C to remove debris. The resulting supernatant, containing total cellular protein, was carefully collected and transferred into fresh microcentrifuge tubes. Protein concentration was quantified by Bicinchoninic Acid assay (Merck, Milan, Italy) according to the manufacturer's instructions.

### 2.8 Superoxide Dismutase (SOD) Activity

SOD activity was determined in organoid lysates using SOD Determination Kit (Merck, Milan, Italy) according to the manufacturer's protocol. The SOD activity levels were normalized to cell protein concentrations. Three technical replicates for each experiment were used.

### 2.9 Lactate Dehydrogenase (LDH) Release Activity

For toxicity evaluation, the release of LDH into the culture media was determined using a Cytotoxicity Detection Kit (Roche, Penzberg, Germany) according to the manufacturer's protocol. The LDH levels were normalized to cell protein concentrations. Three technical replicates for each experiment were used.

### 2.10 RNA Isolation, Reverse Transcription, and Quantitative Polymerase Chain Reaction (qPCR)

Total RNA was isolated from organoids with RNeasy micro kit (Qiagen, Milan, Italy) and 500 ng of RNA was used for retrotranscription by using iScript cDNA Synthesis Kit (Bio‐Rad, Hercules, CA, USA). qPCR was performed using SsoAdvanced Universal SYBR Green Supermix (Bio‐Rad, Hercules, CA, USA) following the thermal profile suggested by the kit. Validated mouse primers for *Ccl2* (qMmuCED0003785), *Pyy* (qMmuCED0001589), *Mki67* (qMmuCID0018717), *Ccnd1* (qMmuCID0023518), *B2m* (qMmuCID0040553), and *Actb* (qMmuCED0027505) were purchased from Bio‐Rad. The calculation of differential expression of the transcripts was performed by the 2^−ΔΔ*C*t^ formula and normalized on *B2m* and *Actb* expression level.

### 2.11 DRG Neurons Immunofluorescence and Imaging

At the conclusion of the treatment, DRG neurons were rinsed with PBS and fixed with 4% paraformaldehyde for 15 min at room temperature. Following fixation, neurons were washed three times with PBS (5 min each) and permeabilized using 0.3% Triton X‐100 in PBS for 10 min at room temperature. Cells were then incubated for 30 min in a blocking solution composed of 0.5% bovine serum albumin and 0.3% Triton X‐100 in PBS. For immunostaining, DRG neurons were incubated overnight at 4°C with the following primary antibodies diluted in blocking solution: rabbit anti‐mouse c‐Fos (1:100; BS‐0469R, Lot AI08094815, RRID: AB_10858019; Bioss Antibodies, Woburn, MA, USA) and goat anti‐mouse CGRP (1:500; PA1‐85250, Lot XL3781521A, RRID: AB_2259435; Invitrogen‐Life Technologies, Milan, Italy). The next day, cells were washed three times in PBS (5 min each) and incubated in the dark for 2 h at room temperature with the secondary antibody donkey anti‐goat IgG Alexa Fluor 568 (1:500; A‐11057, RRID: AB_2534104; Invitrogen‐Life Technologies, Milan, Italy), prepared in blocking solution. After another three PBS washes, neurons were incubated for an additional 2 h in the dark at room temperature with goat anti‐rabbit IgG Alexa Fluor 488 (1:500; A‐11034, RRID: AB_2576217; Invitrogen‐Life Technologies, Milan, Italy). Following final washes (three times, 5 min each with PBS), slides were mounted using Fluoroshield mounting medium with DAPI (Merck, Milan, Italy). Negative controls (without primary antibodies) were included in each immunostaining experiment and processed in parallel. Imaging was performed using a motorized Leica DM6000 B microscope equipped with a DFC350FX digital camera (Leica Microsystems, Mannheim, Germany). For quantitative analysis of c‐Fos and CGRP expression, three ×20 magnification fields per slide were analyzed, with data collected from an average of two to three independent slides per condition. Representative images for the illustrative panel were acquired at ×40 magnification. Fluorescence quantification was carried out using ImageJ software (Schneider et al. 2012). Individual neurons were identified using the region of interest (ROI) manager. Fluorescence intensity was measured for each ROI, and background fluorescence was subtracted for each channel. For each slide, the mean fluorescence intensity of all ROIs was calculated. Final values are reported as the average of means from two to three slides per experimental group.

### 2.12 Visceral Pain Model and Treatment With GMG + MYR

Experimental colitis was induced following previously described methods with slight modifications (Simeoli et al. 2017; Singh et al. 2020). Briefly, mice were given 2.5% (w:v) DSS (AbMole BioScience, Houston, TX, USA) in tap water ad libitum from Day 0 to Day 5. GMG was dissolved in PBS at 10 mL kg^−1^ and, after bioactivation with MYR (23.4 U mL^−1^) at 37°C for 30 min (100 mg kg^−1^ GMG + 250 μL kg^−1^ MYR; 10 mL kg^−1^), administered orally once daily from Day 0 to Day 7 in DSS‐treated animals as a preventive treatment. The doses of GMG (30 and 100 mg kg^−1^) were selected based on previous studies reporting the efficacy and safety of GMG‐derived isothiocyanates in experimental models of inflammation and pain (Martínez‐González et al. 2017; Kim, Jaja‐Chimedza, et al. 2018), as well as to explore a low‐to‐high dose range suitable for evaluating dose‐dependent effects following oral administration. Control mice received only the vehicle. Visceral sensitivity was assessed by CRD using graded balloon volumes, according to previously established protocols (Delprete et al. 2023; Ciampi et al. 2025; Margiotta et al. 2025). This approach has been widely applied in preclinical models to evaluate inflammation‐associated visceral hypersensitivity. Hot plate and Paw Pressure tests were included as complementary measures of secondary somatic hyperalgesia and generalized nociceptive sensitization, which are frequently observed in preclinical models of IBD and visceral hypersensitivity. In addition, the Open Field test was employed to evaluate pain‐associated behavioral alterations and changes in locomotor activity and exploratory behavior, which are commonly affected in conditions of persistent inflammation and nociceptive sensitization (Xia et al. 2022; Aghamiri et al. 2024; Mohammadgholi‐Beiki et al. 2024). Mice were grouped as follows: *n* = 6 veh + veh; *n* = 8 DSS + veh; *n* = 8 DSS + GMG 30 mg kg^−1^ + MYR; *n* = 8 DSS + GMG 100 mg kg^−1^ + MYR.

### 2.13 Assessment of Visceral Sensitivity by Abdominal Withdrawal Reflex (AWR)

CRD‐induced visceral sensitivity was assessed by scoring the AWR in conscious mice, using a validated semiquantitative scale (Delprete et al. 2023). Mice were briefly anesthetized with 2% isoflurane (VIRBAC S.r.l., Milan, Italy) and a lubricated latex balloon attached to polyethylene tubing, constructed from a Fogarty 4F embolectomy catheter (Edwards Lifesciences, Milan, Italy) and connected to a water‐filled syringe, was gently inserted into the rectum and distal colon via the anus. The tubing was secured to the animal's tail to ensure consistent positioning during testing. Following a 30‐min recovery period to allow full regaining of consciousness, AWR responses to graded CRD volumes (50, 100, 150, and 200 μL) were recorded. Observers blinded to treatment conditions evaluated behavioral responses using the following scoring criteria: 0, no response; 1, stillness with occasional head movement at stimulus onset; 2, visible contraction of abdominal muscles without lifting of the abdomen; 3, pronounced abdominal contraction with elevation of the abdomen; 4, intense response including body arching and lifting of pelvic region and scrotum. A minimum interval of 3 min was observed between successive distensions.

### 2.14 Hot Plate Test

Thermal hyperalgesia was evaluated using a Hot Plate (Ugo Basile, Varese, Italy) maintained at 50°C ± 1°C. Mice were placed in a Plexiglas cylinder (diameter 10 cm, height 15 cm) to limit movement, and latency to paw licking or shaking was recorded. A 30‐s cut‐off was used to prevent injury (Lucarini et al. 2025).

### 2.15 Paw Pressure Test

Mechanical nociceptive threshold was assessed with an Analgesimeter (Ugo Basile, Varese, Italy). Increasing mechanical pressure was applied to the dorsal surface of the hind paw until vocalization or withdrawal occurred. Thresholds were reported in grams, with an arbitrary cut‐off of 100 g (Micheli et al. 2021).

### 2.16 Open Field Test

Pain‐associated anxiety‐like behaviors and locomotor activity were assessed in an Open Field arena. Mice were placed at the center and allowed to explore for 10 min while video‐tracking recorded total distance traveled, center entries, and time spent in the center versus corners. The arena was cleaned with 20% ethanol between trials (D'Amato et al. 2020).

### 2.17 Colitis Severity and Macroscopic Colon Damage

The disease activity index (DAI) was used to evaluate colitis severity based on weight loss, fecal blood, and stool consistency (Simeoli et al. 2017; Singh et al. 2020):

– Weight loss: 0 (none); 1 (1%–3%); 2 (3%–6%); 3 (6%–9%); 4 (> 9%).
– Fecal blood: 0 (normal); 2 (visible); 4 (rectal bleeding).
– Stool consistency: 0 (normal); 2 (loose); 4 (diarrhea).

After sacrifice, colons were harvested to measure wall thickness and length, as indicative parameters of intestinal damage.

### 2.18 Histological Analysis of Colon Damage

The presence of colon damage was investigated ex vivo in accordance with the methods used in previous studies (Lucarini et al. 2021). For the histological analysis, the colon was fixed in 4% paraformaldehyde for 24 h, dehydrated in alcohol, included in paraffin and cut into 5 μm sections. The microscopic evaluations of colon damage were carried out on three different hematoxylin and eosin (H&E)‐stained sections per animal by two blind investigators. The microscopic damage was scored in accordance with the criteria reported previously (Lucarini et al. 2020): mucosal architecture loss (0–3); goblet cell depletion (0, absent; 1, present); crypt abscess (0, absent; 1, present); cellular infiltration (0–3); *tunica muscularis* thickening (0–3). Representative digitalized images were collected by a Leica DMRB light microscope equipped with a DFC480 digital camera (×4 and ×10 objective, ×40 and ×100 magnification; Leica Microsystems, Mannheim, Germany).

### 2.19 Statistical Analysis

All experiments were conducted using randomized allocation of samples and animals to experimental groups. Investigators were blinded to treatment conditions during data acquisition and analysis for all the experiments. Group sizes (*n*) are reported in the corresponding figure legends and refer to biological replicates. Results are expressed as mean ± SEM. The analysis of variance was performed by one‐way ANOVA with Bonferroni's sie54m' gnificant difference procedure used for post hoc comparisons. Results from qPCR analysis are displayed as box and whiskers plot (median, percentiles and percentiles + 1.5 interquantile range), with analysis of variance performed by Kruskal–Wallis test followed by Dunn's test for post hoc comparisons. *p* values < 0.05 were considered significant. Data were analyzed using OriginPro 10.1.5.132 software (OriginLab).

## 3 Results

### 3.1 Efficacy of MOR in Reducing CKs‐Induced Inflammatory Damage in Colon Organoids

**FIGURE 1:**
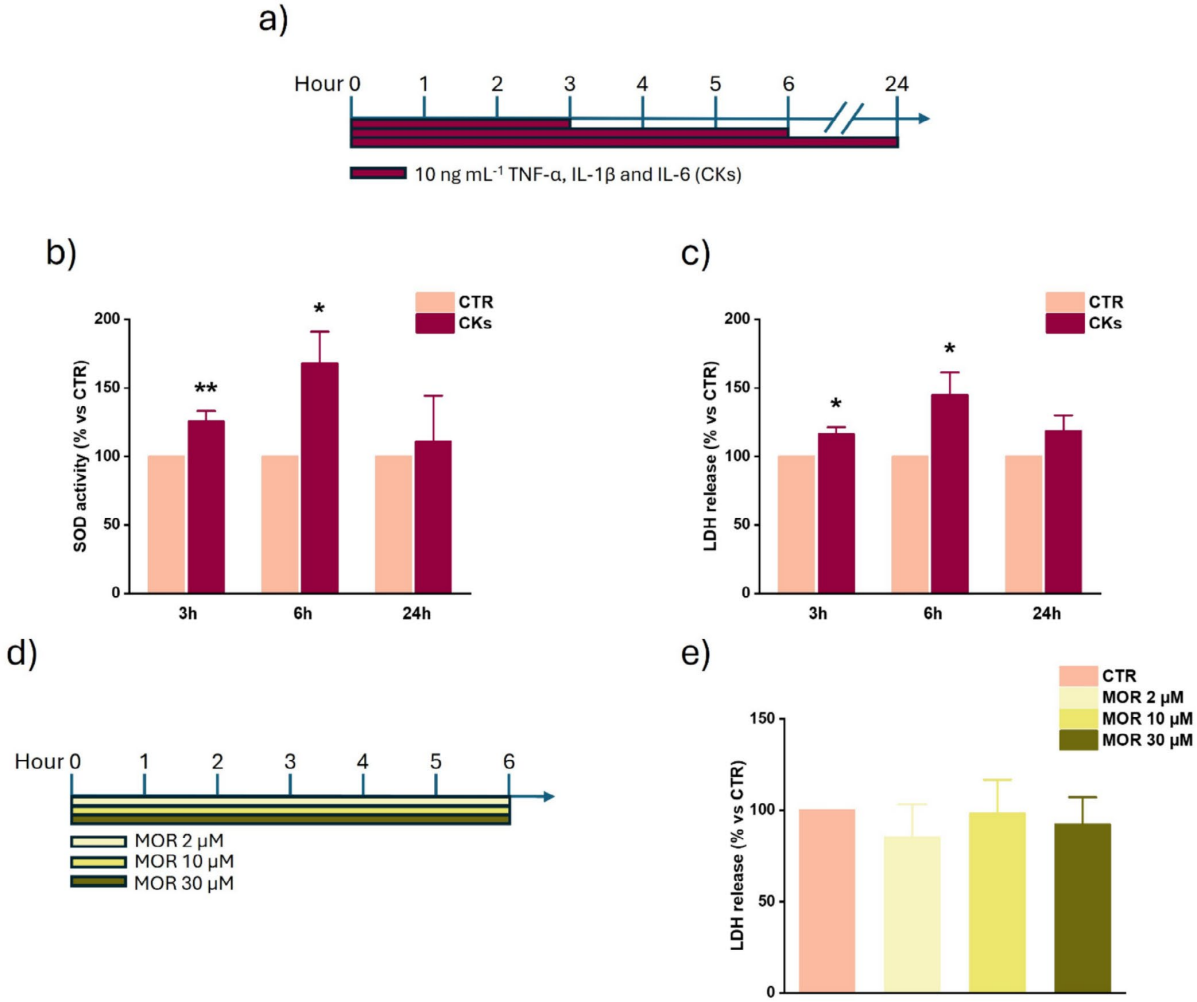
Effects of CKs and MOR on colon organoids. (a) Mature organoids were treated with 10 ng mL^−1^ of TNF‐α, IL‐1β, and IL‐6 for 3, 6 and 24 h. (b) SOD and (c) LDH release assays were performed to evaluate CKs cytotoxicity and pro‐oxidant effects on colon organoids. Data are represented as mean ± SEM of *n* = 3–4 experiments. (d) Mature organoids were treated with MOR 2–30 μM for 6 h. (e) LDH release assay was performed to evaluate MOR cytotoxicity on colon organoids. Data are represented as mean ± SEM of *n* = 2 experiments. *p < 0.05, **p < 0.01 versus CTR.

Colon organoids were previously characterized (Margiotta et al. 2025). We exposed colon organoids to a mix of CKs consisting of TNF‐α (10 ng mL^−1^), IL‐1β (10 ng mL^−1^), and IL‐6 (10 ng mL^−1^) for 3, 6, and 24 h (Figure 1a). After 3 h of treatment with CKs, organoids displayed a slight increase in oxidative stress levels (Figure 1b) and LDH release (Figure 1c). However, the greatest increase in both parameters was observed after 6 h of CKs treatment, while after 24 h, LDH release and oxidative stress values dropped to control levels. Thus, the best time for treating colon organoids with CKs was 6 h, and further analysis was carried out at this timepoint. Based on these results, the possible toxicity of MOR on colon organoids was evaluated by treating them with MOR 2–30 μM for 6 h (Figure 1d). LDH release assay showed no toxic effects for any of the three doses used (Figure 1e).

**FIGURE 2:**
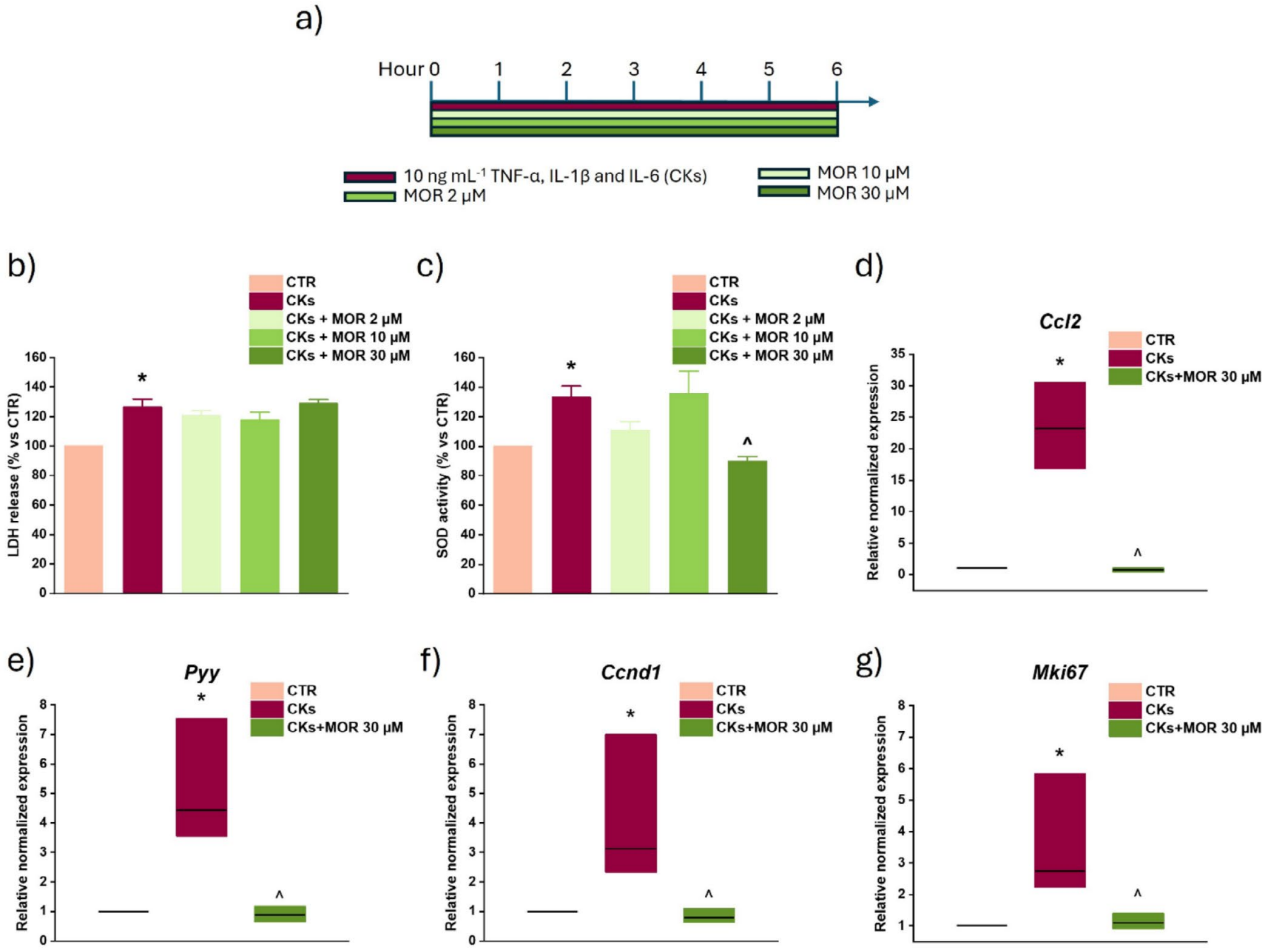
Effects of MOR on colon organoids treated with CKs. (a) Mature organoids were treated with 10 ng mL^−1^ of TNF‐α, IL‐1β and IL‐6 + MOR 2–30 μM for 6 h. (b) LDH release and (c) SOD assays were performed to evaluate MOR effects on cytotoxicity and oxidative stress induced by CKs on colon organoids. Data are represented as mean ± SEM of *n* = 2–3 experiments. (d–g) qPCR was performed to evaluate MOR effects on the upregulation of inflammation‐related genes induced by CKs in colon organoids. Data are represented as box and whiskers of *n* = 3 experiments (median, 25th and 75th percentiles, and 25th and 75th percentiles +1.5 interquantile range). *p < 0.05 versus CTR; ^*p* < 0.05 versus CKs.

Then, we assessed the ability of MOR 2, 10, and 30 μM to counteract the cytotoxicity increase induced by CKs in colon organoids by treating them simultaneously with CKs and MOR for 6 h (Figure 2a). As shown in Figure 2b, no dose of MOR was able to reduce the cytotoxicity associated with CKs treatment. Anyway, MOR 30 μM was able to fully revert the increased levels of oxidative stress in CKs‐treated organoids (Figure 2c). Therefore, MOR 30 μM was tested to reduce the expression of genes related to inflammatory processes in organoids exposed to CKs. qPCR analysis showed that MOR 30 μM significantly decreased the expression of *Ccl2*, *Pyy*, *Ccnd1*, and *Mki67* that were strongly upregulated in the CKs group, restoring their expression to control levels (Figure 2d–g).

### 3.2 Efficacy of MOR in Reducing Inflammation‐Induced Rise of Pain‐Related Markers in DRG Neurons via Its Bioactivity on Intestinal Epithelium

**FIGURE 3:**
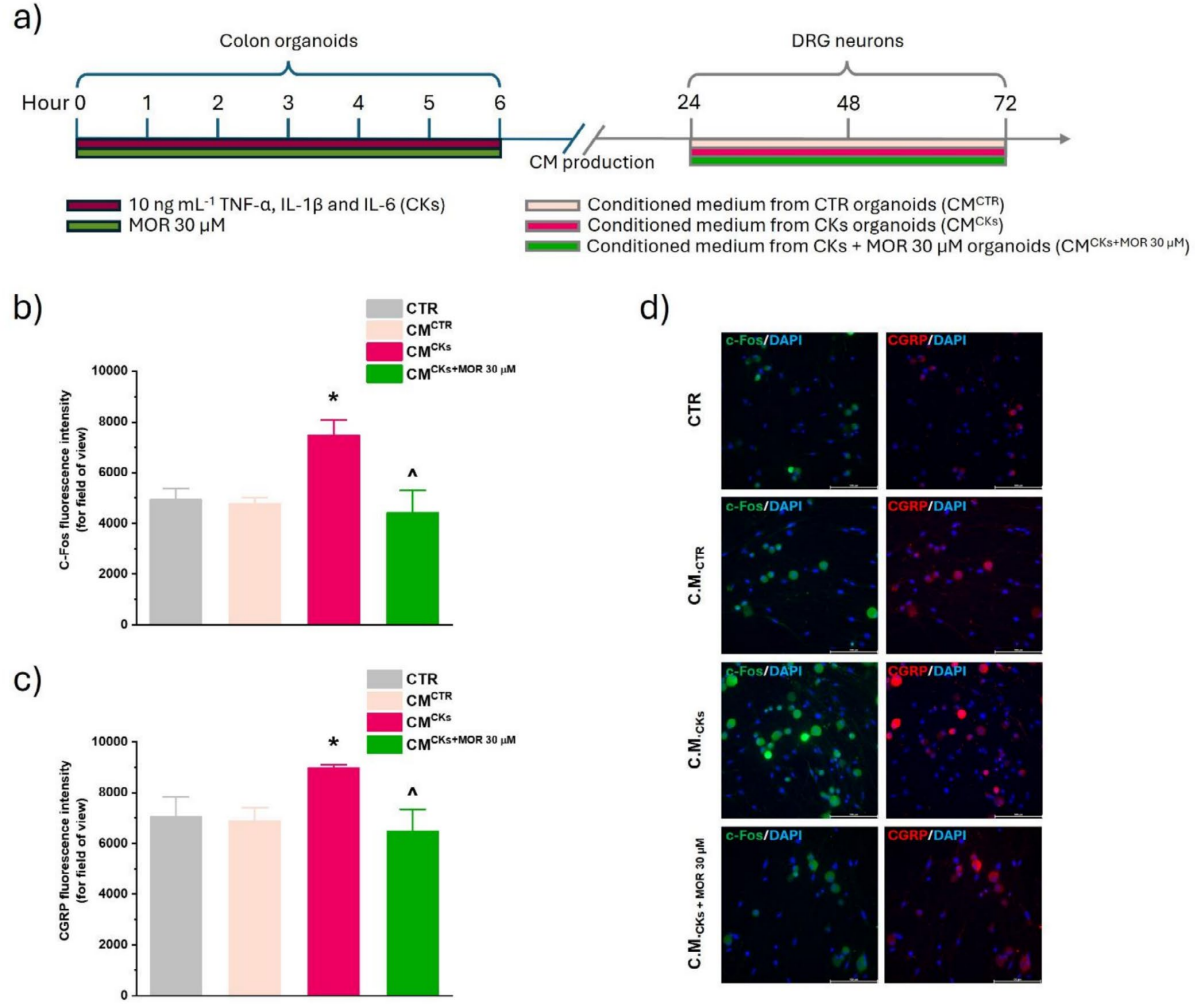
Effects of MOR in preventing c‐Fos and CGRP increase in DRG neurons exposed to conditioned media from inflamed intestinal organoids. (a) Conditioned media from 6‐h CKs‐treated (CM^CKs^) and CKs + MOR 30 μM‐treated (CM^CKs + MOR 30 μM^) organoids were used to treat DRG neurons for 48 h. Fluorescence intensity was measured for (b) c‐Fos and (c) CGRP in all experimental groups. (d) Representative images of DRG neurons after exposure to all the experimental conditions (magnification: ×40; scale bar: 100 μm). Data are represented as mean ± SEM of *n* = 2–3 different slides for each condition. *p < 0.05 versus CM^CTR^; ^*p* < 0.05 versus CM^CKs^.

To further investigate the efficacy of MOR in fighting intestinal inflammation and related visceral hypersensitivity, we tested whether it could modulate neuronal response to inflamed gut epithelium via its bioactivity on the epithelium itself. To reproduce the intestinal epithelial–neuronal signaling, DRG neurons were exposed for 48 h to CM from CTR organoids (CM^CTR^), CKs‐treated organoids (CM^CKs^), and CKs + MOR 30 μM‐treated organoids (CM^CKs + MOR 30 μM^), as shown in Figure 3a. Exposure to CM^CKs^ induced increased fluorescence intensity for c‐Fos and CGRP in DRG neurons, while CM^CKs + MOR 30 μM^ prevented such increment (Figure 3b–d). These data suggest that the presence of MOR in conditions of intestinal inflammation counteracts the rise of neuronal activation and pain‐related markers by reducing alterations of the intestinal epithelium. The choice of c‐Fos and CGRP as readouts was based on their established roles as complementary markers of neuronal activation and nociceptive signaling, respectively. c‐Fos is a widely used immediate early gene rapidly induced by extracellular stimuli and neuronal activation (Chung 2015), while CGRP is a neuropeptide selectively expressed by nociceptive sensory neurons (Iyengar et al. 2017) and critically involved in visceral pain transmission (Schou et al. 2017; Noor‐Mohammadi et al. 2021). Notably, in a previous study from our group employing a comparable epithelial–neuronal in vitro model, changes in c‐Fos and CGRP expression were shown to coincide with direct electrophysiological measures of neuronal activation, including increased neuronal excitability (Margiotta et al. 2025). This supports the interpretation of these molecular markers as reliable indicators of pain‐related neuronal activation in response to gut‐derived inflammatory signals, even in the absence of direct functional measurements in the present study.

### 3.3 Efficacy of Bioactivated GMG in Counteracting DSS‐Induced Colitis and Pain in Mice

**FIGURE 4:**
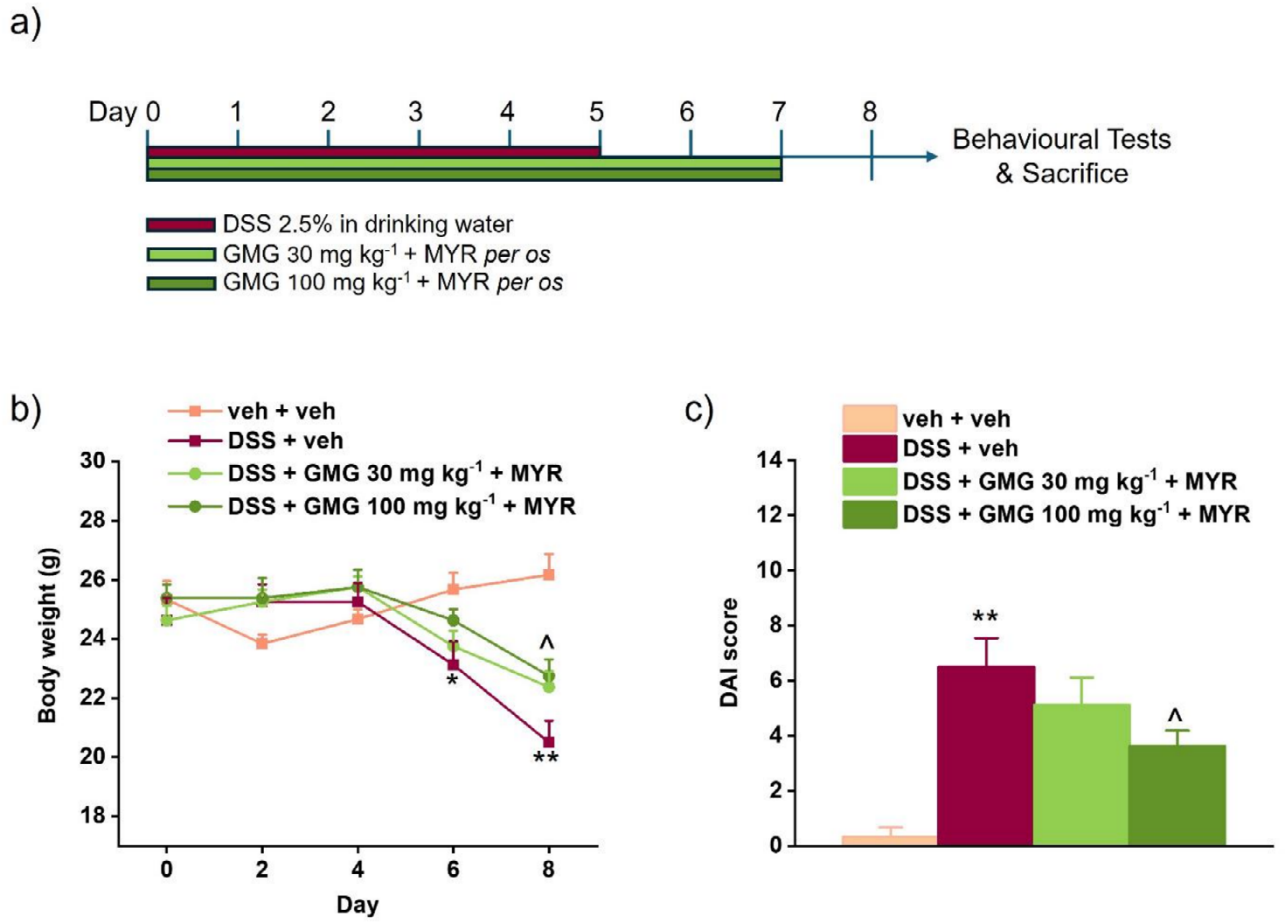
Effect of bioactivated glucomoringin (GMG + MYR) supplementation on the disease activity index of DSS‐treated mice. (a) Mice received DSS 2.5% in drinking water starting at Day 0 for 5 days and bioactivated GMG (30–100 mg kg^−1^ + MYR) was administered orally once daily from Day 0 to Day 7 in DSS‐treated animals. (b) Body weight was monitored every 2 days starting at Day 0. (c) On Day 8, following behavioral tests, DAI scores were assigned based on weight loss, stool consistency, and rectal bleeding. Data are represented as mean ± SEM (6–8 mice per group). *p < 0.05, **p < 0.01 versus veh + veh; ^*p* < 0.05 versus DSS + veh.

In the in vivo study, we evaluated the analgesic and intestinal protective effects of MYR‐activated GMG, the glucosinolate precursor of MOR, in a murine DSS‐induced colitis model. GMG was pre‐incubated with MYR to produce a fresh solution of MOR isothiocyanate. Mice received daily oral doses of GMG + MYR for 7 days, beginning on Day 0 concurrently with DSS treatment, as reported in the experimental scheme (Figure 4a). At the end of the treatment, DAI components, weight loss, fecal blood, and stool consistency were scored. Behavioral testing was performed on Day 8, followed by sacrifice to assess intestinal damage. Visceral hypersensitivity was measured by AWR to CRD, while somatic hypersensitivity to mechanical and thermal stimuli was assessed via Paw Pressure and Hot Plate tests. Behavioral changes (mobility and exploration) were analyzed using Open Field assays. GMG + MYR mitigated DSS‐induced weight loss (Figure 4b), improved stool consistency and reduced rectal bleeding, leading to a significant decrease in DAI score (Figure 4c).

**FIGURE 5:**
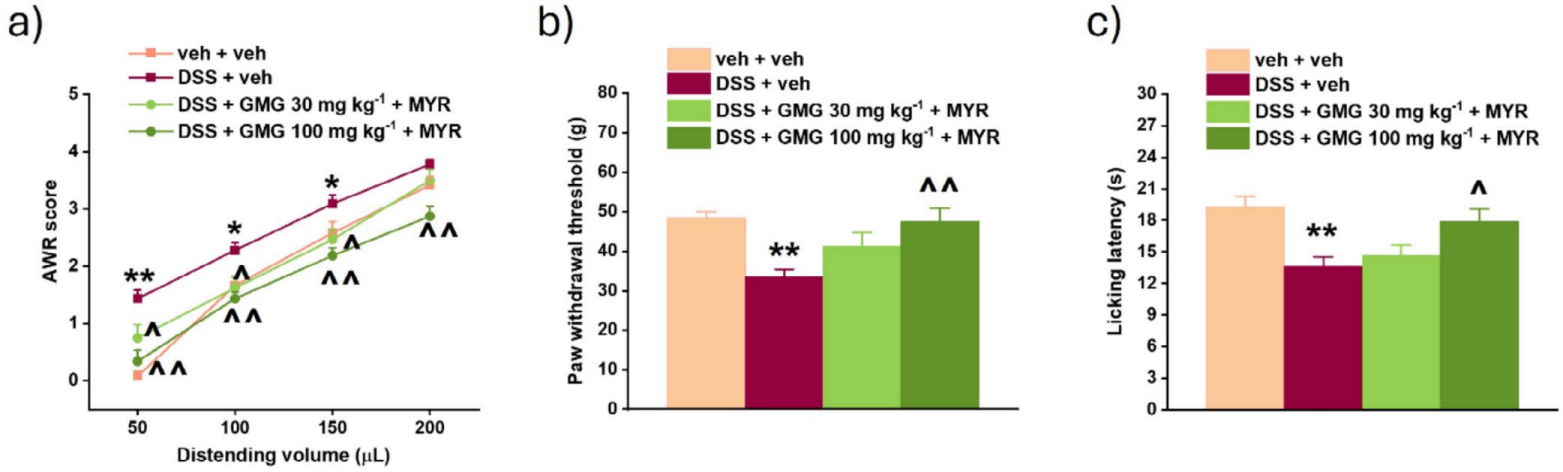
Antihyperalgesic efficacy of bioactivated glucomoringin (GMG + MYR) on visceral and somatic pain in DSS‐treated mice. On Day 8, 24 h after the last GMG + MYR dose, (a) AWR response to colorectal distension, (b) mechanical hyperalgesia via Paw Pressure test, and (c) thermal hyperalgesia via Hot Plate test were assessed. Data are represented as mean ± SEM (6–8 mice per group). *p < 0.05, **p < 0.01 versus veh + veh; ^*p* < 0.05, ^^*p* < 0.01 versus DSS + veh.

DSS treatment significantly increased AWR scores across all distension volumes compared to control (veh + veh), while GMG + MYR treatment (30–100 mg kg^−1^ + MYR) dose‐dependently reduced visceral hypersensitivity (Figure 5a); the lowest dose was partially effective, while the highest dose fully prevented DSS‐induced abdominal pain, restoring AWR scores to control levels. Concomitantly, GMG 100 mg kg^−1^ + MYR attenuated mechanical and thermal hyperalgesia (Paw Pressure, Figure 5b; Hot Plate, Figure 5c).

**FIGURE 6:**
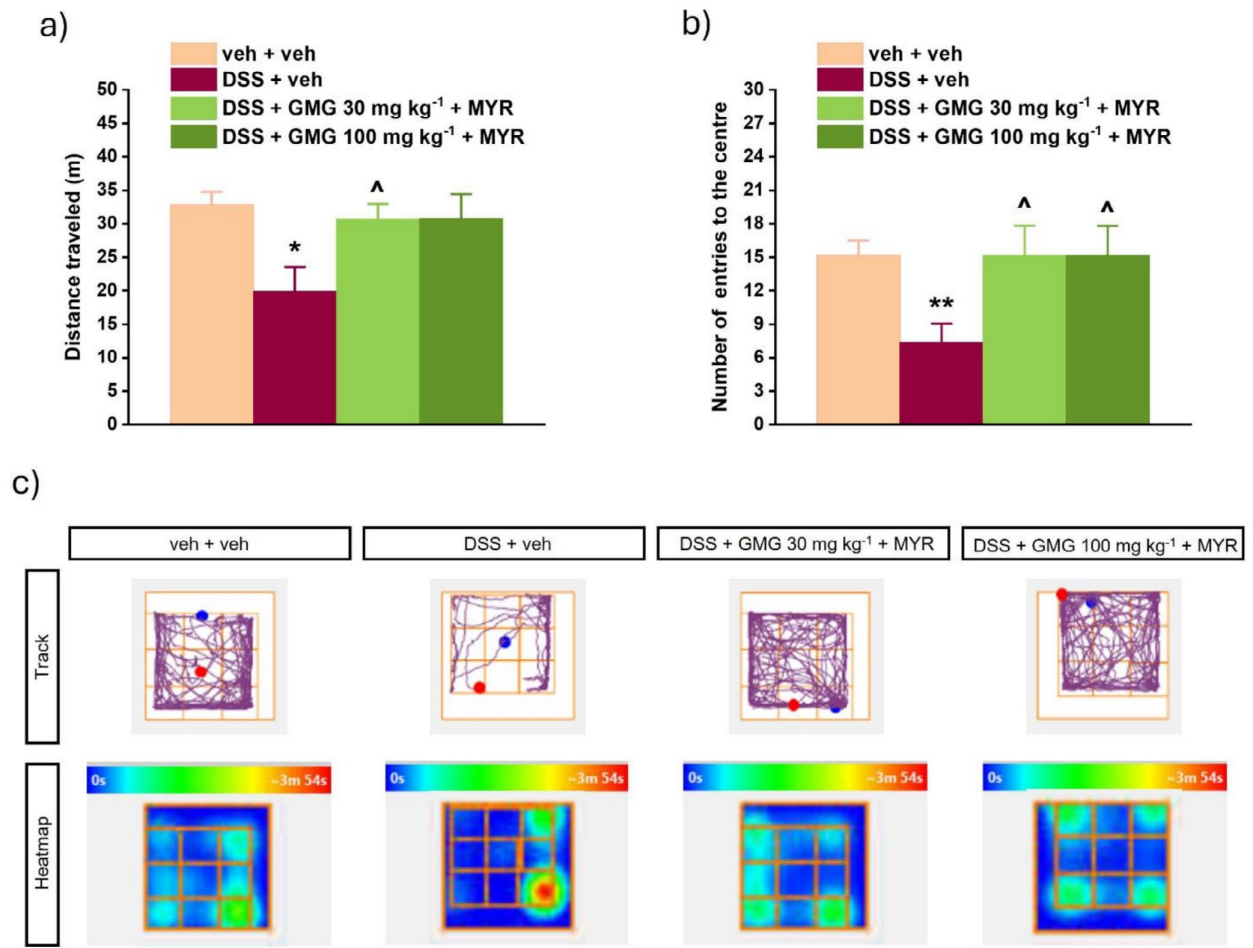
Effect of bioactivated glucomoringin (GMG + MYR) on pain‐related behavioral changes in DSS‐treated mice. On Day 8, 24 h after final GMG + MYR dose, Open Field test evaluated: (a) total distance traveled, (b) center entries, (c) representative Track and Heatmap. Data are represented as mean ± SEM (6–8 mice per group). *p < 0.05, **p < 0.01 versus veh + veh; ^*p* < 0.05 versus DSS + veh.

Moreover, mice given DSS + GMG 100 mg kg^−1^ + MYR exhibited significantly improved behavior in the Open Field, as demonstrated by the greater total distance traveled (Figure 6a) and more entries in the center of arena (Figure 6b) than DSS + vehicle, indicating reduced anxiety‐like behavior and preserved exploratory drive. These results are well represented in Figure 6c, showing the track and the heatmap of animals in the Open Field test.

### 3.4 Protective Role of Bioactivated GMG on Inflammatory Damage to Colon Induced by DSS

**FIGURE 7:**
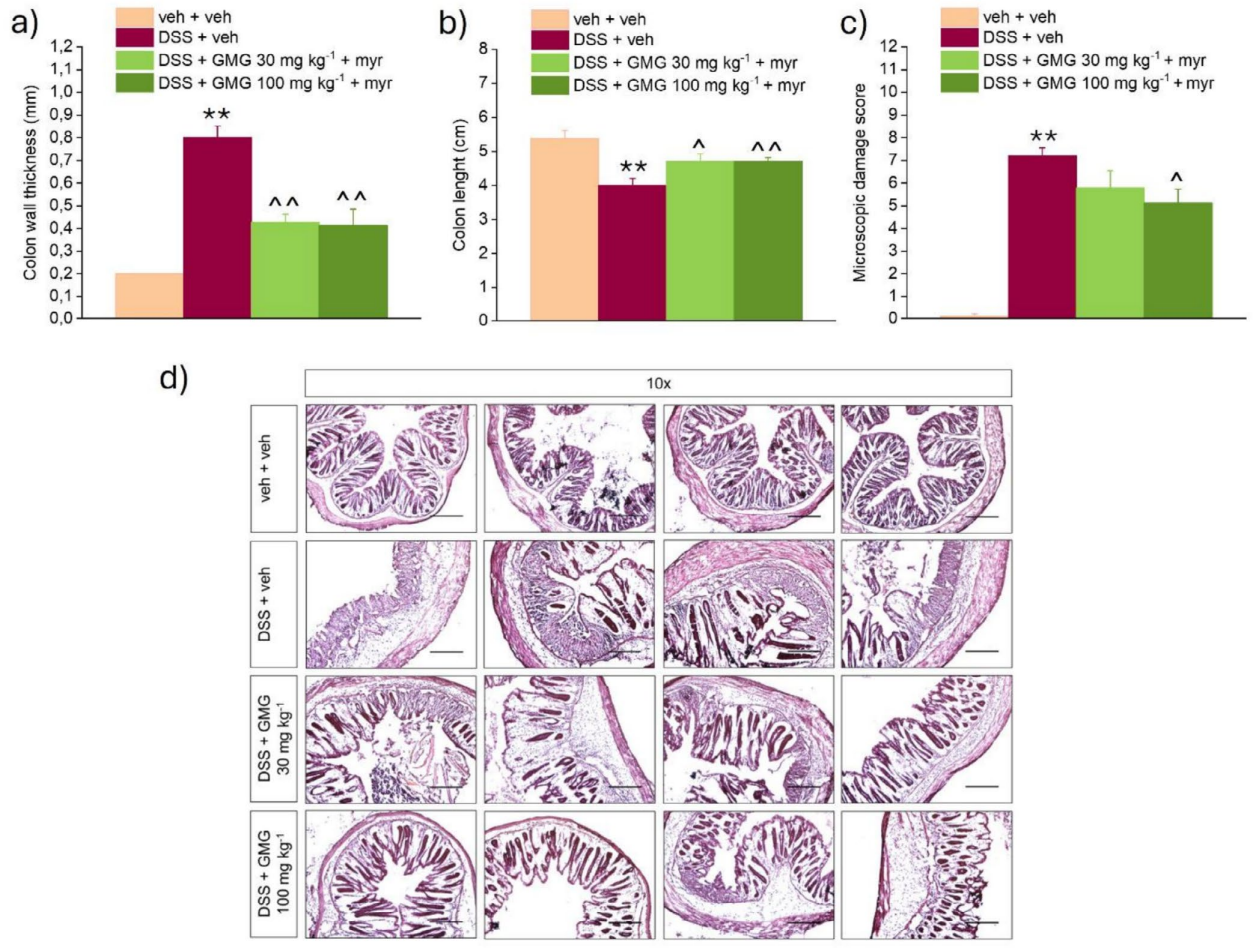
Protective effect of bioactivated glucomoringin (GMG + MYR) on DSS‐induced colonic damage. Animals were sacrificed after completing behavioral tests and tissues collected for histological analysis. (a) Colon thickness and (b) length were measured on freshly harvested colon, while (c) microscopic damage was assessed on H&E‐stained sections. (d) Representative images of H&E‐stained sections are shown to highlight inflammatory cell infiltration, crypt architecture, goblet cell depletion, and epithelial integrity (×100 magnification; scale bar: 200 μm). Data are represented as mean ± SEM (6–8 mice per group). **p < 0.01 versus veh + veh; ^*p* < 0.05, ^^*p* < 0.01 versus DSS + veh.

Colon tissue damage was evaluated macroscopically, by measuring colon thickness and length (Figure 7a,b), and microscopically, by histological analysis on H&E‐stained slices (Figures 7c,d and S1). Macroscopic evaluation confirmed GMG + MYR protective effect: both 30 and 100 mg kg^−1^ doses significantly reduced colon wall thickening (Figure 7a) and prevented colon shortening (Figure 7b) caused by DSS. Histological examination of H&E‐stained colonic sections from DSS‐treated mice revealed pronounced inflammatory damage. Key features included diffuse submucosal edema, substantial infiltration of inflammatory cells, thickening of the *muscularis propria*, and extensive mucosal injury. Notably, there was crypt elongation and degeneration, a marked reduction in goblet cell numbers and the presence of ulcerative lesions, consistent with established characteristics of DSS‐induced colitis. Administration of GMG + MYR at a dose of 100 mg kg^−1^ markedly attenuated DSS‐induced colonic injury. This treatment preserved mucosal architecture, reduced submucosal edema, and substantially decreased inflammatory cell infiltration. In contrast, the lower 30 mg kg^−1^ dose of GMG + MYR, while offering partial protection, did not fully restore normal colonic histology: notable mucosal irregularities persisted. These findings suggest a dose‐dependent therapeutic efficacy of GMG + MYR in ameliorating histopathological features of DSS‐induced colitis (Figure 7c,d) and reducing the extent of tissue damage, as shown in an overview of tissue damage (Figure S1).

## 4 Discussion

In this work, we employed a multifaceted approach to prove the efficacy of purified MOR and bioactivated GMG (GMG + MYR) in counteracting intestinal epithelial damage and visceral hypersensitivity under inflammatory conditions typical of IBD. Through a combined system consisting of colon organoids and DRG neurons we proved the efficacy of MOR in both reducing intestinal epithelial stress and inflammation‐induced rise of neuronal activation and pain‐related markers. Results were validated in vivo, ensuring the analgesic and anti‐inflammatory effects of the bioactivated precursor GMG with MYR in a DSS‐induced colitis model.

Abdominal pain is a prevalent and debilitating symptom in IBD patients, representing a major unmet clinical challenge due to its complex pathophysiology and poor responsiveness to conventional analgesics (Ford et al. 2024). Current therapeutic strategies are therefore primarily directed at controlling mucosal inflammation and preventing disease progression rather than providing effective visceral analgesia (Barberio et al. 2023; Massironi et al. 2024; Fudman et al. 2025). Alarmingly, a substantial proportion of patients (20%–50%) continue to experience abdominal pain even during phases of clinical and endoscopic remission (Trikola 2025), underscoring the persistent clinical need for targeting analgesia in gastrointestinal disorders, beyond the primary disease.

Herbal medicine was found to be effective in managing abdominal pain given the multiple mechanisms of action which work synergistically to address its complex pathophysiology (Lashgari et al. 2024). Several studies reported that *M. oleifera* and its phytoconstituents have shown a broad range of biological activities, such as anti‐microbial, anti‐inflammatory, antioxidant, neuroprotective and analgesic activities (Galuppo et al. 2013; Galuppo, Giacoppo, Iori, De Nicola, Milardi, et al. 2015; Rajan et al. 2016; Jaafaru et al. 2018; Pareek et al. 2023). Few studies investigated *M. oleifera* efficacy against inflammatory damage to the colon, revealing protective effects by regulating pro‐inflammatory pathways (Zhang, Cao, et al. 2024; Zhang, Zhao, et al. 2024) and modulating gut microbiota (Husien et al. 2024). Anti‐inflammatory activity of *M. oleifera* was also described in murine intestinal organoids exposed to TNF‐α (Zhang, Cao, et al. 2024). Nevertheless, no one investigated its efficacy against chronic visceral pain caused by colitis. It has been described that *M. oleifera* extracts significantly reduced acetic acid‐induced abdominal contortions in rodents (Adedapo et al. 2015; Palomino‐Pacheco et al. 2024). However, the acetic acid injection fails to meet established validity criteria for the abdominal pain model, as it mainly reflects acute peritoneal irritation rather than the multifactorial mechanisms underlying chronic visceral hypersensitivity (Ness 1999).

Colon organoids are an innovative platform to study the role of intestinal epithelium, providing improved reliability of results compared to cell lines (Tian et al. 2023) and an advanced tool to investigate the epithelium crosstalk with other cells in different pathological conditions. This is particularly relevant in the context of IBD, where the epithelial compartment plays a central role in the onset and persistence of chronic inflammation (Martini et al. 2017), as well as in the mechanisms underlying visceral hypersensitivity (Najjar et al. 2020; Margiotta et al. 2023, 2025). At the same time, they are an excellent screening system, being a faster and easier study platform than animal models, also considering the ethical complications of their use. Yet, the possibility to obtain them from human samples strengthens the translational potential of preclinical results.

In this view, we used a dual in vivo and in vitro approach to demonstrate the value of complex in vitro models for identifying a promising epithelial‐targeting mechanism and screening new therapies for visceral pain conditions beyond the treatment of inflammatory disease. Since IL‐1β, IL‐6, and TNF‐α are the major CKs involved in intestinal inflammation in IBD conditions (Strober and Fuss 2011; Adak and Khan 2018; Aebisher et al. 2025), to establish the in vitro model of colonic inflammation we exposed murine colon organoids to 10 ng mL^−1^ of IL‐1β, IL‐6, and TNF‐α up to 24 h. This CKs mixture was already used to induce an inflammatory damage to intestinal organoids (d'Aldebert et al. 2020; Xing et al. 2023) but with different exposure times or concentrations. In particular, we identified 6 h of exposure as the highest peak of damage in terms of oxidative stress and cytotoxicity. Starting from the evidence that MOR (2–30 μM) incubation for 6 h did not affect organoids' viability, we assessed its efficacy in counteracting CKs damage. Although MOR (2–30 μM) was not able to effectively mitigate cytotoxicity caused by CKs as measured by LDH release, the highest concentration markedly attenuated oxidative damage to colon organoids, confirming the antioxidant potential of this compound. CKs and ROS are tightly interconnected, each influencing the other and jointly contributing to the pathogenesis of various diseases, such as IBD (Sun et al. 2024). Specifically, TNF‐α, IL‐1β, and IL‐6 trigger signaling cascades that promote ROS generation via enzymes such as NADPH oxidase and mitochondrial pathways (Lambeth 2004; Bhol et al. 2024). ROS, in turn, can activate transcription factors such as NF‐κB, thereby sustaining additional CK production, inflammation, and apoptosis (Bhol et al. 2024). Noteworthy, NF‐κB pathways and subsequent cell injury can also be directly activated by CKs (Guo et al. 2024). Thus, the observed dissociation between antioxidant effects and the lack of protection against CKs‐induced LDH release could hypothetically reflect a selective modulation of NF‐κB signaling, whereby MOR attenuates ROS‐driven NF‐κB activation but does not interfere with CK‐mediated activation. Pro‐inflammatory CKs, particularly TNF‐α, are known to activate epithelial cell death programs such as extrinsic apoptosis via caspase‐8 activation, as well as regulate necrotic pathways including necroptosis mediated by RIPK1/RIPK3/MLKL signaling (Vandenabeele et al. 2010; Pasparakis and Vandenabeele 2015). These forms of CK‐driven cell death have been implicated in intestinal epithelial damage during colitis and may hypothetically not be readily reversible by antioxidant interventions alone (Akanyibah et al. 2024). It is therefore plausible that, at the selected experimental timepoint, a subpopulation of epithelial cells undergoes irreversible damage following CK challenge and is no longer functionally recoverable, despite effective attenuation of intracellular oxidative stress in the remaining viable cells. In particular, intestinal stem cells and Paneth cells have been identified as highly CK‐sensitive populations whose loss can disproportionately contribute to epithelial damage and barrier dysfunction during intestinal inflammation (Günther et al. 2011; Saito et al. 2021). It is therefore conceivable that the LDH release observed in our organoid model originates predominantly from a subpopulation of severely compromised epithelial cells that are intrinsically more susceptible to CK‐induced cell death and thus less amenable to rescue by antioxidant interventions. In contrast, MOR may preferentially preserve redox homeostasis and functional integrity in more resilient epithelial populations, resulting in a measurable reduction of oxidative stress without a complete prevention of CK‐induced cytotoxicity at the organoid level. This finding is in line with previous evidence attesting MOR regulation of NRF2 and NF‐κB pathways in response to lipopolysaccharide‐driven sepsis and inflammation (Sailaja et al. 2021). NRF2/KEAP1 pathway is a major regulator of the antioxidant defense systems of cells, modulating the expression of a plethora of genes related to redox balance (Wang et al. 2024). NRF2/KEAP1 and NF‐κB regulation is shared with other naturally occurring isothiocyanates, such as sulforaphane (4*R*_*S*_‐(methylsulfinyl)butyl isothiocyanate) (Shah et al. 2024) and erucin (4‐(methylsulfanyl)butyl isothiocyanate) (Wagner et al. 2015), that are found to be effective in different forms of chronic pain (Lucarini, Micheli, Di Cesare Mannelli, et al. 2022). Importantly, such selective epithelial protection may still be sufficient to modulate epithelial–neuronal signaling and contribute to the attenuation of visceral hypersensitivity.

MOR 30 μM was also found to be effective in reducing the gene expression of key factors involved in inflammation, such as *Ccl2*, *Pyy, Ccnd1*, and *Mki67*, significantly increased after the exposure of colon organoids to CKs. Noteworthy, chemokine (C‐C motif) ligand 2 (CCL2) is a chemokine released by gut epithelium that attracts macrophages and monocytes to the inflamed intestinal mucosa, thereby promoting intestinal inflammation (Singh et al. 2021). Elevated levels of CCL2 were observed in mucosa and sera of IBD patients (Reinecker et al. 1995; Singh et al. 2016), and targeting this pathway showed therapeutic potential by reducing immune cell infiltration (Hachiya et al. 2021). Peptide YY (PYY), produced by enteroendocrine cells, mainly affects appetite and digestion (El‐Salhy et al. 2020). We interpret the increase of *Pyy* expression in CKs‐treated organoids as a compensatory mechanism aimed at limiting inflammation‐induced damage. This observation comes from experimental data showing that PYY expression is elevated in the colonic mucosa of animals with experimental colitis, where exogenous administration of PYY has been reported to exert protective effects against mucosal injury (Li et al. 2022). By contrast, IBD patients displayed a reduced density of PYY‐positive enteroendocrine cells in the colon (El‐Salhy et al. 2013), suggesting that the regulation of PYY during inflammation may be context‐ and stage‐dependent, with an initial upregulation that could be lost during chronic disease. The normalization of PYY expression upon MOR treatment in our system suggests that this compound modulates inflammation‐driven enteroendocrine responses, contributing to the maintenance of epithelial homeostasis. Organoids also underwent an upregulation of *Ccnd1* and *Mki67* following CKs exposure, providing evidence that inflammatory stimuli foster epithelial proliferation and trigger cell cycle activation (Kondo et al. 2021; Khoramjoo et al. 2022; Mobbs et al. 2024; Zhang, Cao, et al. 2024). Notably, MOR treatment restored the expression of both markers toward baseline, further indicating its capacity to modulate inflammation‐induced epithelial responses.

The ability of MOR to protect the intestinal epithelium against inflammation was reflected in preventing the rise of pain‐related markers in DRG neurons exposed to epithelial‐derived factors. Neurons cultured with CM^CKs^ showed increased c‐Fos and CGRP production compared to those cultured with CM^CTR^ or CM^CKs + MOR 30 μM^. Thus MOR, acting as a brake on the pro‐excitatory signaling traveling from the epithelium to neurons under inflammatory conditions, showed indirect neuromodulatory effects. While the use of epithelial CM allowed us to isolate and demonstrate the contribution of epithelium‐derived soluble factors to sensory neuron modulation, this approach does not capture potential contact‐dependent signaling between epithelial cells and neurons, which could be further explored in future studies using direct co‐culture or microphysiological models. Moreover, although the present study does not include direct functional assessments of neuronal activity, the interpretation of increased c‐Fos and CGRP expression as markers of pain‐related neuronal activation is supported by previous evidence. In particular, we have recently demonstrated that gut‐derived inflammatory signals capable of inducing c‐Fos and CGRP upregulation in DRG neurons also produce parallel increases in neuronal excitability assessed by electrophysiological recordings in a comparable in vitro system (Margiotta et al. 2025). These findings strengthen the mechanistic link between epithelial inflammation, neuronal activation, and visceral pain signaling, and support the validity of the molecular readouts used in the current work.

To validate in vitro results, both protection on gut epithelium and anti‐hyperalgesic effect exerted by *M. oleifera* chemical tags were further investigated in the mouse model of visceral pain associated with DSS‐induced colitis. In this case, we used the MOR precursor GMG due to its higher solubility in water. GMG was preactivated with MYR to improve the bioavailability of MOR, a bioactivation approach already reported for GMG and other glucosinolates such as thiofunctionalyzed glucoerucin (4‐(methylsulfanyl)butyl glucosinolate) and glucoraphanin (4*R*_*S*_‐(methylsulfinyl)butyl glucosinolate) (Wagner et al. 2015; Fahey, Wade, Stephenson, Panjwani, et al. 2019; Lucarini, Micheli, Pagnotta, et al. 2022; Razis et al. 2025). We demonstrated that oral daily treatment with hydrolyzed GMG + MYR (30–100 mg kg^−1^ + MYR) exerts dose‐dependent anti‐hyperalgesic effects in mice. The use of multiple pain endpoints reflects the complex interplay between persistent peripheral visceral inflammation and centrally mediated cross‐sensitization, whereby sustained nociceptive input from the inflamed gut promotes referred somatic hypersensitivity and broader alterations in nociceptive processing. This concept is supported by preclinical literature showing that inflammatory bowel pathology can be associated with both visceral hypersensitivity and altered somatic nociception (Xia et al. 2022; Aghamiri et al. 2024; Mohammadgholi‐Beiki et al. 2024). In this context, the present findings not only corroborate the efficacy of GMG + MYR across multiple pain‐related readouts, but also open the way for future studies employing additional paradigms, such as assessments of spontaneous pain or complementary tests of anxiety‐related behavior, to further refine and extend the evaluation of compound effects on pain‐associated behaviors.

Moreover, the improvements in DAI and histological integrity of the colon confirm that MOR/GMG + MYR can protect from inflammatory damage to the colon, consistent with results coming from the in vitro model. This is in line with a recent work showing MOR efficacy against colitis (Zhang, Zhao, et al. 2024). Similarly, other isothiocyanate compounds, such as allyl isothiocyanate, have been shown to ameliorate DSS‐induced colitis severity by influencing barrier integrity and reducing inflammation (Kim, Choi, et al. 2018). Our results extend these reports by demonstrating that MOR/GMG + MYR exert multifaceted protective actions, not only by attenuating inflammation and preserving epithelial integrity, but also by modulating neurosensory pathways implicated in visceral hypersensitivity and related behavioral alterations, which are frequent readouts of pain and possibly anxiety‐like states (Bushnell et al. 2013; De La Rosa et al. 2024). Moreover, beyond strengthening the current literature on the topic, these findings establish a solid foundation for advancing alternatives to animal studies in the preclinical screening of novel therapies for colitis‐associated pain. By capturing key aspects of epithelium–neuron interactions, our in vitro approach provides a mechanistically informed framework to anticipate the therapeutic efficacy of interventions targeting pain onset following inflammatory insult. This work builds on a recently published strategy to investigate dysbiosis‐related visceral hypersensitivity in vitro (Margiotta et al. 2025) further underscoring the value of this approach both for advancing pathophysiological insights and for facilitating drug screening. While additional pharmacokinetic and toxicological studies are necessary to establish the efficacy, safety profile, and optimal dosage regimen of the investigated compound in patients, the 100 mg kg^−1^ dose administered in mice lies within a range commonly used in preclinical studies and may support future dose‐scaling considerations.

Furthermore, although molecular analyses of colonic and neuronal tissues were not performed in the in vivo experiments, the consistency between the mechanistic effects observed in vitro and the functional, behavioral, and histopathological outcomes detected in vivo supports the proposed epithelial‐protective and neuromodulatory mechanism of action of GMG + MYR. In particular, the reduction of oxidative stress and inflammatory signaling in intestinal organoids, together with the attenuation of epithelial‐driven expression of neuronal activation and pain‐related markers, parallels the marked protection against colonic damage and visceral hypersensitivity observed in the DSS model. Future investigations incorporating tissue‐specific molecular readouts in vivo will be valuable to further dissect the cellular pathways underlying these effects.

Importantly, it should be considered that MOR may exert its beneficial effects on pain and pain‐related comorbidities through mechanisms extending beyond the protection of the gut epithelium and the modulation of the epithelial–neuronal crosstalk. Indeed, this compound could directly influence pain pathways, acting at multiple levels to regulate nociceptive processing. Supporting this hypothesis, MOR has already been reported as effective in neuropathic pain (Giacoppo et al. 2017), suggesting that its analgesic activity is not restricted to visceral hypersensitivity and inflammatory conditions. Such evidence points to a broader pharmacological profile, in which the compound may simultaneously target inflammatory cascades, oxidative stress, and neuronal excitability, ultimately contributing to its multimodal protective and analgesic actions. A limitation of this study is the exclusive use of male mice. Given known sex differences in pain perception and inflammatory responses, future studies will be required to determine whether MOR/GMG + MYR exerts comparable effects in female animals. Furthermore, no validated pharmacological positive control was included, as there are currently no universally accepted therapies that selectively and reliably target visceral pain mechanisms in IBD. Inclusion of available agents would not have been mechanistically comparable to the epithelium‐centered approach of the present study. Future studies may benefit from incorporating emerging, mechanism‐specific modulators of epithelial–neuronal communication as they become available.

## 5 Conclusion

In conclusion, this study demonstrates that MOR and its bioactivated precursor GMG + MYR exert multifaceted protective effects in experimental colitis, ranging from the preservation of intestinal epithelial homeostasis to the attenuation of inflammation‐associated visceral pain. By integrating a reductionist in vitro epithelial–neuronal model with in vivo functional and histopathological validation, our work highlights the relevance of epithelial‐driven mechanisms in shaping pain outcomes under inflammatory conditions.

Importantly, this combinatorial strategy supports the use of advanced organoid‐based platforms as valuable tools to inform and guide preclinical drug discovery, while reducing reliance on animal experimentation. Together, these findings provide a robust framework for the development and mechanistic evaluation of multitarget interventions aimed at mitigating intestinal inflammation and its associated pain symptomatology.

## Author Contributions

**Francesco Margiotta:** conceptualization, data curation, formal analysis, investigation, methodology, validation, visualization, writing – original draft, writing – review and editing. **Elena Lucarini:** conceptualization, data curation, formal analysis, investigation, methodology, validation, visualization, writing – original draft, writing – review and editing. **Alessandra Toti:** conceptualization, data curation, formal analysis, investigation, methodology, validation, visualization. **Maria Giovanna Cataldi:** investigation, methodology. **Clara Ciampi:** investigation, validation, visualization. **Gina Rosalinda De Nicola:** methodology, writing – review and editing. **Lorenzo Di Cesare Mannelli:** conceptualization, funding acquisition, project administration, supervision, writing – review and editing. **Carla Ghelardini:** conceptualization, funding acquisition, project administration, supervision.

## Funding

This work was supported by the European Union—Next Generation EU—National Recovery and Resilience Plan, Mission 4 Component 2—Investment 1.5—THE—Tuscany Health Ecosystem—(ECS00000017 to C.G.)—CUP B83C22003920001; European Union—Next Generation EU—National Recovery and Resilience Plan, Mission 4 Component 2—Investment 1.4—Strengthening research facilities and creation of “Campioni Nazionali di R&S” on Key Enabling Technologies—National Center for Gene Therapy and Drugs based on RNA Technology—(CN00000041 to L.D.C.M)—CUP B13C22001010001.; and Italian Ministry of University and Research (MUR)—“Dipartimenti di Eccellenza 2023‐2027”—[58514_DIPECC_23_27 to the Department NEUROFARBA].

## Conflicts of Interest

The authors declare no conflicts of interest.

## Supporting information


**Figure S1:** Protective effect of bioactivated glucomoringin (GMG + MYR) on DSS‐induced colonic damage. Representative images of H&E‐stained sections (×40 magnification; scale bar: 500 μm) are shown to allow the visualization of the entire colonic section and ensure a representative overview of tissue damage. For each image, the respective inserts (×100 magnification; scale bar: 200 μm) highlight inflammatory cell infiltration, crypt architecture, goblet cell depletion, and epithelial integrity.

## Data Availability

The data underlying this article are available in Mendeley Data, at https://doi.org/10.17632/5jbdzw78br.1.

## References

[ptr70303-bib-0001] Adak, A. , and M. R. Khan . 2018. “An Insight Into Gut Microbiota and Its Functionalities.” Cellular and Molecular Life Sciences: CMLS 76: 473–493. 30317530 10.1007/s00018-018-2943-4 PMC11105460

[ptr70303-bib-0002] Adedapo, A. A. , O. O. Falayi , and A. A. Oyagbemi . 2015. “Evaluation of the Analgesic, Anti‐Inflammatory, Anti‐Oxidant, Phytochemical and Toxicological Properties of the Methanolic Leaf Extract of Commercially Processed *Moringa oleifera* in Some Laboratory Animals.” Journal of Basic and Clinical Physiology and Pharmacology 26: 491–499. 26020553 10.1515/jbcpp-2014-0105

[ptr70303-bib-0003] Aebisher, D. , D. Bartusik‐Aebisher , A. Przygórzewska , P. Oleś , P. Woźnicki , and A. Kawczyk‐Krupka . 2025. “Key Interleukins in Inflammatory Bowel Disease—A Review of Recent Studies.” International Journal of Molecular Sciences 26: 121. 10.3390/ijms26010121 PMC11719876 39795980

[ptr70303-bib-0004] Aghamiri, H. , A. Mohammadgholi‐Beiki , R. Rashidian , et al. 2024. “Zhumeria Majdae Essential Oil Attenuates TNBS‐Induced Colitis in Rats by Regulating Inflammatory and Apoptotic Pathways.” Inflammopharmacology 32: 3809–3824. 39312096 10.1007/s10787-024-01574-0

[ptr70303-bib-0005] Akanyibah, F. A. , Y. Zhu , T. Jin , D. K. W. Ocansey , F. Mao , and W. Qiu . 2024. “The Function of Necroptosis and Its Treatment Target in IBD.” Mediators of Inflammation 2024: 7275309. 39118979 10.1155/2024/7275309 PMC11306684

[ptr70303-bib-0006] Aldebert, E. d.’ , M. Quaranta , M. Sébert , et al. 2020. “Characterization of Human Colon Organoids From Inflammatory Bowel Disease Patients.” Frontiers in Cell and Developmental Biology 8: 363. 32582690 10.3389/fcell.2020.00363 PMC7287042

[ptr70303-bib-0007] Barbara, G. , M. R. Barbaro , D. Fuschi , et al. 2021. “Inflammatory and Microbiota‐Related Regulation of the Intestinal Epithelial Barrier.” Frontiers in Nutrition 8: 718356. 34589512 10.3389/fnut.2021.718356 PMC8475765

[ptr70303-bib-0008] Barberio, B. , D. J. Gracie , C. J. Black , and A. C. Ford . 2023. “Efficacy of Biological Therapies and Small Molecules in Induction and Maintenance of Remission in Luminal Crohn's Disease: Systematic Review and Network Meta‐Analysis.” Gut 72: 264–274. 35907636 10.1136/gutjnl-2022-328052

[ptr70303-bib-0009] Bayrer, J. R. , J. Castro , A. Venkataraman , et al. 2023. “Gut Enterochromaffin Cells Drive Visceral Pain and Anxiety.” Nature 616: 137–142. 36949192 10.1038/s41586-023-05829-8 PMC10827380

[ptr70303-bib-0010] Bhol, N. K. , M. M. Bhanjadeo , A. K. Singh , et al. 2024. “The Interplay Between Cytokines, Inflammation, and Antioxidants: Mechanistic Insights and Therapeutic Potentials of Various Antioxidants and Anti‐Cytokine Compounds.” Biomedicine & Pharmacotherapy 178: 117177. 39053423 10.1016/j.biopha.2024.117177

[ptr70303-bib-0011] Borgonovo, G. , L. De Petrocellis , A. Schiano Moriello , et al. 2020. “Moringin, A Stable Isothiocyanate From *Moringa oleifera*, Activates the Somatosensory and Pain Receptor TRPA1 Channel In Vitro.” Molecules 25: 976. 32098328 10.3390/molecules25040976 PMC7070407

[ptr70303-bib-0012] Bushnell, M. C. , M. Čeko , and L. A. Low . 2013. “Cognitive and Emotional Control of Pain and Its Disruption in Chronic Pain.” Nature Reviews. Neuroscience 14: 502–511. 23719569 10.1038/nrn3516 PMC4465351

[ptr70303-bib-0013] Chassaing, B. , J. D. Aitken , M. Malleshappa , and M. Vijay‐Kumar . 2014. “Dextran Sulfate Sodium (DSS)‐Induced Colitis in Mice.” Current Protocols in Immunology 104: 15.25.1–15.25.14. 10.1002/0471142735.im1525s104 PMC3980572 24510619

[ptr70303-bib-0014] Chung, L. 2015. “A Brief Introduction to the Transduction of Neural Activity Into Fos Signal.” Development & Reproduction 19: 61–67. 27004262 10.12717/DR.2015.19.2.061 PMC4801051

[ptr70303-bib-0015] Ciampi, C. , F. Fagiani , V. Murtaj , et al. 2025. “Intestinal Inflammation Induces Glymphatic Remodeling, Priming Early Neurodegenerative Signals in Male Mice.” Alzheimer's & Dementia 21: e70640. 10.1002/alz.70640 PMC12509038 41065132

[ptr70303-bib-0016] D'Amato, A. , L. Di Cesare Mannelli , E. Lucarini , et al. 2020. “Faecal Microbiota Transplant From Aged Donor Mice Affects Spatial Learning and Memory via Modulating Hippocampal Synaptic Plasticity‐ and Neurotransmission‐Related Proteins in Young Recipients.” Microbiome 8: 140. 33004079 10.1186/s40168-020-00914-w PMC7532115

[ptr70303-bib-0017] De La Rosa, J. S. , B. R. Brady , M. M. Ibrahim , et al. 2024. “Co‐Occurrence of Chronic Pain and Anxiety/Depression Symptoms in U.S. Adults: Prevalence, Functional Impacts, and Opportunities.” Pain 165: 666–673. 37733475 10.1097/j.pain.0000000000003056 PMC10859853

[ptr70303-bib-0018] Delprete, C. , R. R. Giorgini , E. Lucarini , et al. 2023. “Disruption of the Microbiota‐Gut‐Brain Axis Is a Defining Characteristic of the α‐Gal A (−/0) Mouse Model of Fabry Disease.” Gut Microbes 15: 2256045. 37712629 10.1080/19490976.2023.2256045 PMC10506438

[ptr70303-bib-0019] Di Giorgio, C. , R. Roselli , M. Biagioli , et al. 2023. “Modeling Inflammatory Bowel Disease by Intestinal Organoids.” Recent Advances in Inflammation & Allergy Drug Discovery 17: 39–53. 36411558 10.2174/2772270817666221121143853

[ptr70303-bib-0020] du Sert, N. P. , A. Ahluwalia , S. Alam , et al. 2020. “Reporting Animal Research: Explanation and Elaboration for the ARRIVE Guidelines 2.0.” PLoS Biology 18: e3000411. 32663221 10.1371/journal.pbio.3000411 PMC7360025

[ptr70303-bib-0021] El‐Salhy, M. , J. G. Hatlebakk , and T. Hausken . 2020. “Possible Role of Peptide YY (PYY) in the Pathophysiology of Irritable Bowel Syndrome (IBS).” Neuropeptides 79: 101973. 31727345 10.1016/j.npep.2019.101973

[ptr70303-bib-0022] El‐Salhy, M. , T. Mazzawi , D. Gundersen , J. G. Hatlebakk , and T. Hausken . 2013. “The Role of Peptide YY in Gastrointestinal Diseases and Disorders (Review).” International Journal of Molecular Medicine 31: 275–282. 23292145 10.3892/ijmm.2012.1222 PMC4042877

[ptr70303-bib-0023] Fahey, J. W. , K. L. Wade , K. K. Stephenson , et al. 2019. “Bioavailability of Sulforaphane Following Ingestion of Glucoraphanin‐Rich Broccoli Sprout and Seed Extracts With Active Myrosinase: A Pilot Study of the Effects of Proton Pump Inhibitor Administration.” Nutrients 11: 1489. 31261930 10.3390/nu11071489 PMC6682992

[ptr70303-bib-0024] Fahey, J. W. , K. L. Wade , K. K. Stephenson , et al. 2019. “A Strategy to Deliver Precise Oral Doses of the Glucosinolates or Isothiocyanates From *Moringa oleifera* Leaves for Use in Clinical Studies.” Nutrients 11: 1547. 31323988 10.3390/nu11071547 PMC6682957

[ptr70303-bib-0025] Fan, Y.‐Y. , L. A. Davidson , and R. S. Chapkin . 2019. “Murine Colonic Organoid Culture System and Downstream Assay Applications.” Methods in Molecular Biology 1576: 171–181. 27539462 10.1007/7651_2016_8 PMC5316509

[ptr70303-bib-0026] Flood, P. , N. Hanrahan , K. Nally , and S. Melgar . 2024. “Human Intestinal Organoids: Modeling Gastrointestinal Physiology and Immunopathology—Current Applications and Limitations.” European Journal of Immunology 54: e2250248. 37957831 10.1002/eji.202250248

[ptr70303-bib-0027] Ford, A. C. , S. Vanner , P. C. Kashyap , and Y. Nasser . 2024. “Chronic Visceral Pain: New Peripheral Mechanistic Insights and Resulting Treatments.” Gastroenterology 166: 976–994. 38325759 10.1053/j.gastro.2024.01.045 PMC11102851

[ptr70303-bib-0028] Fudman, D. I. , R. A. McConnell , C. Ha , and S. Singh . 2025. “Modern Advanced Therapies for Inflammatory Bowel Diseases: Practical Considerations and Positioning.” Clinical Gastroenterology and Hepatology 23: 454–468. 39147217 10.1016/j.cgh.2024.06.050 PMC12180935

[ptr70303-bib-0029] Galuppo, M. , S. Giacoppo , R. Iori , G. R. De Nicola , P. Bramanti , and E. Mazzon . 2015. “Administration of 4‐(α‐L‐Rhamnosyloxy)‐Benzyl Isothiocyanate Delays Disease Phenotype in SOD1(G93A) Rats: A Transgenic Model of Amyotrophic Lateral Sclerosis.” BioMed Research International 2015: 259417. 26075221 10.1155/2015/259417 PMC4436451

[ptr70303-bib-0030] Galuppo, M. , S. Giacoppo , R. Iori , et al. 2015. “4(α‐l‐Rhamnosyloxy)‐Benzyl Isothiocyanate, a Bioactive Phytochemical That Defends Cerebral Tissue and Prevents Severe Damage Induced by Focal Ischemia/Reperfusion.” Journal of Biological Regulators and Homeostatic Agents 29: 343–356. 26122222

[ptr70303-bib-0031] Galuppo, M. , G. R. D. Nicola , R. Iori , P. Dell'utri , P. Bramanti , and E. Mazzon . 2013. “Antibacterial Activity of Glucomoringin Bioactivated With Myrosinase Against Two Important Pathogens Affecting the Health of Long‐Term Patients in Hospitals.” Molecules 18: 14340–14348. 24264136 10.3390/molecules181114340 PMC6270176

[ptr70303-bib-0032] Giacoppo, S. , R. Iori , P. Bramanti , and E. Mazzon . 2017. “Topical Moringin‐Cream Relieves Neuropathic Pain by Suppression of Inflammatory Pathway and Voltage‐Gated Ion Channels in Murine Model of Multiple Sclerosis.” Molecular Pain 13: 1744806917724318. 28741431 10.1177/1744806917724318 PMC5555508

[ptr70303-bib-0033] Günther, C. , E. Martini , N. Wittkopf , et al. 2011. “Caspase‐8 Regulates TNF‐α‐Induced Epithelial Necroptosis and Terminal Ileitis.” Nature 477: 335–339. 21921917 10.1038/nature10400 PMC3373730

[ptr70303-bib-0034] Guo, Q. , Y. Jin , X. Chen , et al. 2024. “NF‐κB in Biology and Targeted Therapy: New Insights and Translational Implications.” Signal Transduction and Targeted Therapy 9: 53. 38433280 10.1038/s41392-024-01757-9 PMC10910037

[ptr70303-bib-0035] Hachiya, K. , M. Masuya , N. Kuroda , et al. 2021. “Irbesartan, an Angiotensin II Type 1 Receptor Blocker, Inhibits Colitis‐Associated Tumourigenesis by Blocking the MCP‐1/CCR2 Pathway.” Scientific Reports 11: 19943. 34620946 10.1038/s41598-021-99412-8 PMC8497524

[ptr70303-bib-0036] Hashash, J. G. , J. Elkins , J. D. Lewis , and D. G. Binion . 2024. “AGA Clinical Practice Update on Diet and Nutritional Therapies in Patients With Inflammatory Bowel Disease: Expert Review.” Gastroenterology 166: 521–532. 38276922 10.1053/j.gastro.2023.11.303

[ptr70303-bib-0037] Husien, H. M. , S. U. Rehman , Z. Duan , and M. Wang . 2024. “Effect of *Moringa oleifera* Leaf Polysaccharide on the Composition of Intestinal Microbiota in Mice With Dextran Sulfate Sodium‐Induced Ulcerative Colitis.” Frontiers in Nutrition 11: 1409026. 38765820 10.3389/fnut.2024.1409026 PMC11099247

[ptr70303-bib-0038] ISO . 2019. “ISO 9167:2019.”

[ptr70303-bib-0039] Iyengar, S. , M. H. Ossipov , and K. W. Johnson . 2017. “The Role of Calcitonin Gene‐Related Peptide in Peripheral and Central Pain Mechanisms Including Migraine.” Pain 158: 543–559. 28301400 10.1097/j.pain.0000000000000831 PMC5359791

[ptr70303-bib-0040] Jaafaru, M. S. , N. A. Abd Karim , M. E. Enas , P. Rollin , E. Mazzon , and A. F. Abdull Razis . 2018. “Protective Effect of Glucosinolates Hydrolytic Products in Neurodegenerative Diseases (NDDs).” Nutrients 10: 580. 29738500 10.3390/nu10050580 PMC5986460

[ptr70303-bib-0041] Jaja‐Chimedza, A. , B. L. Graf , C. Simmler , et al. 2017. “Biochemical Characterization and Anti‐Inflammatory Properties of an Isothiocyanate‐Enriched Moringa ( *Moringa oleifera* ) Seed Extract.” PLoS One 12: e0182658. 28792522 10.1371/journal.pone.0182658 PMC5549737

[ptr70303-bib-0042] Keefer, L. , J. G. Hashash , E. Szigethy , and E. A. Mayer . 2024. “AGA Clinical Practice Update on Pain Management in Inflammatory Bowel Disease: Commentary.” Gastroenterology 166: 1182–1189. 38639677 10.1053/j.gastro.2024.03.034

[ptr70303-bib-0043] Khoramjoo, S. M. , N. Kazemifard , S. Baradaran Ghavami , et al. 2022. “Overview of Three Proliferation Pathways (Wnt, Notch, and Hippo) in Intestine and Immune System and Their Role in Inflammatory Bowel Diseases (IBDs).” Frontiers in Medicine 9: 865131. 35677821 10.3389/fmed.2022.865131 PMC9170180

[ptr70303-bib-0044] Kim, M. W. , S. Choi , S. Y. Kim , Y. S. Yoon , J.‐H. Kang , and S. H. Oh . 2018. “Allyl Isothiocyanate Ameliorates Dextran Sodium Sulfate‐Induced Colitis in Mouse by Enhancing Tight Junction and Mucin Expression.” International Journal of Molecular Sciences 19: 2025. 30002285 10.3390/ijms19072025 PMC6073867

[ptr70303-bib-0045] Kim, Y. , A. Jaja‐Chimedza , D. Merrill , O. Mendes , and I. Raskin . 2018. “A 14‐Day Repeated‐Dose Oral Toxicological Evaluation of an Isothiocyanate‐Enriched Hydro‐Alcoholic Extract From *Moringa oleifera* Lam. Seeds in Rats.” Toxicology Reports 5: 418–426. 29854612 10.1016/j.toxrep.2018.02.012 PMC5977371

[ptr70303-bib-0046] Kim, Y. , A. G. Wu , A. Jaja‐Chimedza , et al. 2017. “Isothiocyanate‐Enriched Moringa Seed Extract Alleviates Ulcerative Colitis Symptoms in Mice.” PLoS One 12: e0184709. 28922365 10.1371/journal.pone.0184709 PMC5602518

[ptr70303-bib-0047] Klemm, N. , and S. Moosavi . 2024. “Chronic Abdominal Pain in Patients With Inflammatory Bowel Disease in Remission: A Continuing Challenge for Clinicians.” Digestive Diseases and Sciences 69: 4336–4346. 39537891 10.1007/s10620-024-08716-y

[ptr70303-bib-0048] Kondo, A. , S. Ma , M. Y. Y. Lee , et al. 2021. “Highly Multiplexed Image Analysis of Intestinal Tissue Sections in Patients With Inflammatory Bowel Disease.” Gastroenterology 161: 1940–1952. 34529988 10.1053/j.gastro.2021.08.055 PMC8606000

[ptr70303-bib-0049] Lambeth, J. D. 2004. “NOX Enzymes and the Biology of Reactive Oxygen.” Nature Reviews. Immunology 4: 181–189. 10.1038/nri1312 15039755

[ptr70303-bib-0050] Lapointe, T. K. , and C. Altier . 2011. “The Role of TRPA1 in Visceral Inflammation and Pain.” Channels 5: 525–529. 21993194 10.4161/chan.5.6.18016 PMC3265800

[ptr70303-bib-0051] Lashgari, N.‐A. , N. M. Roudsari , S. Momtaz , et al. 2024. “Systematic Review on Herbal Preparations for Controlling Visceral Hypersensitivity in Functional Gastrointestinal Disorders.” Current Pharmaceutical Biotechnology 25: 1632–1650. 38258770 10.2174/0113892010261502231102040149

[ptr70303-bib-0052] Li, Z. , X. Kuang , T. Chen , T. Shen , and J. Wu . 2022. “Peptide YY 3–36 Attenuates Trinitrobenzene Sulfonic Acid‐Induced Colitis in Mice by Modulating Th1/Th2 Differentiation.” Bioengineered 13: 10144–10158. 35443853 10.1080/21655979.2022.2064147 PMC9161959

[ptr70303-bib-0053] Lucarini, E. , L. Micheli , L. Di Cesare Mannelli , and C. Ghelardini . 2022. “Naturally Occurring Glucosinolates and Isothiocyanates as a Weapon Against Chronic Pain: Potentials and Limits.” Phytochemistry Reviews 21: 647–665.

[ptr70303-bib-0054] Lucarini, E. , L. Micheli , E. Pagnotta , et al. 2022. “The Efficacy of *Camelina sativa* Defatted Seed Meal Against Colitis‐Induced Persistent Visceral Hypersensitivity: The Relevance of PPAR α Receptor Activation in Pain Relief.” Nutrients 14: 3137. 35956313 10.3390/nu14153137 PMC9370738

[ptr70303-bib-0055] Lucarini, E. , E. Pagnotta , L. Micheli , et al. 2025. “Benefits of *Camelina sativa* Supplementation in Morphine Treatment: Enhanced Analgesia, Delayed Tolerance and Reduced Gut Side Effects Through PPAR‐α Receptor Engagement.” International Journal of Molecular Sciences 26: 2519. 40141162 10.3390/ijms26062519 PMC11942378

[ptr70303-bib-0056] Lucarini, E. , C. Parisio , J. J. V. Branca , et al. 2020. “Deepening the Mechanisms of Visceral Pain Persistence: An Evaluation of the Gut‐Spinal Cord Relationship.” Cells 9: 1772. 32722246 10.3390/cells9081772 PMC7464824

[ptr70303-bib-0057] Lucarini, E. , L. Seguella , M. Vincenzi , et al. 2021. “Role of Enteric Glia as Bridging Element Between Gut Inflammation and Visceral Pain Consolidation During Acute Colitis in Rats.” Biomedicine 9: 1671. 10.3390/biomedicines9111671 PMC8616000 34829900

[ptr70303-bib-0058] Margiotta, F. , L. Di Cesare Mannelli , A. Morabito , C. Ghelardini , and E. Lucarini . 2023. “Investigating Epithelial‐Neuronal Signaling Contribution in Visceral Pain Through Colon Organoid‐Dorsal Root Ganglion Neuron Co‐Cultures.” Neural Regeneration Research 19: 1199–1200. 37905863 10.4103/1673-5374.386403 PMC11467916

[ptr70303-bib-0059] Margiotta, F. , E. Lucarini , A. Toti , et al. 2025. “Gut Microbiota Dysbiosis Affects Intestinal Sensitivity Through Epithelium‐To‐Neuron Signaling: Novel Insights From a Colon Organoid‐Based Model to Improve Visceral Pain Therapy.” Gut Microbes 17: 2547029. 40903878 10.1080/19490976.2025.2547029 PMC12413070

[ptr70303-bib-0060] Martínez‐González, C. L. , L. Martínez , E. J. Martínez‐Ortiz , et al. 2017. “ *Moringa oleifera*, a Species With Potential Analgesic and Anti‐Inflammatory Activities.” Biomedicine & Pharmacotherapy 87: 482–488. 28073097 10.1016/j.biopha.2016.12.107

[ptr70303-bib-0061] Martini, E. , S. M. Krug , B. Siegmund , M. F. Neurath , and C. Becker . 2017. “Mend Your Fences: The Epithelial Barrier and Its Relationship With Mucosal Immunity in Inflammatory Bowel Disease.” Cellular and Molecular Gastroenterology and Hepatology 4: 33–46. 28560287 10.1016/j.jcmgh.2017.03.007 PMC5439240

[ptr70303-bib-0062] Massironi, S. , F. Furfaro , S. Bencardino , M. Allocca , and S. Danese . 2024. “Immunity in Digestive Diseases: New Drugs for Inflammatory Bowel Disease Treatment—Insights From Phase II and III Trials.” Journal of Gastroenterology 59: 761–787. 38980426 10.1007/s00535-024-02130-x PMC11339122

[ptr70303-bib-0063] Micheli, L. , R. Rajagopalan , E. Lucarini , et al. 2021. “Pain Relieving and Neuroprotective Effects of Non‐Opioid Compound, DDD‐028, in the Rat Model of Paclitaxel‐Induced Neuropathy.” Neurotherapeutics 18: 2008–2020. 34312766 10.1007/s13311-021-01069-8 PMC8608957

[ptr70303-bib-0064] Mobbs, C. L. , N. J. Darling , and S. Przyborski . 2024. “An In Vitro Model to Study Immune Activation, Epithelial Disruption and Stromal Remodelling in Inflammatory Bowel Disease and Fistulising Crohn's Disease.” Frontiers in Immunology 15: 1357690. 38410518 10.3389/fimmu.2024.1357690 PMC10894943

[ptr70303-bib-0065] Mohammadgholi‐Beiki, A. , M. Sheibani , M. Jafari‐Sabet , M. Motevalian , and P. Rahimi‐Moghaddam . 2024. “Anti‐Inflammatory and Protective Effects of Aripiprazole on TNBS‐Induced Colitis and Associated Depression in Rats: Role of Kynurenine Pathway.” International Immunopharmacology 133: 112158. 38691917 10.1016/j.intimp.2024.112158

[ptr70303-bib-0066] Najjar, S. A. , B. M. Davis , and K. M. Albers . 2020. “Epithelial‐Neuronal Communication in the Colon: Implications for Visceral Pain.” Trends in Neurosciences 43: 170–181. 31983457 10.1016/j.tins.2019.12.007 PMC7047572

[ptr70303-bib-0067] Najjar, S. A. , L. L. Ejoh , E. Loeza‐Alcocer , et al. 2021. “Optogenetic Inhibition of the Colon Epithelium Reduces Hypersensitivity in a Mouse Model of Inflammatory Bowel Disease.” Pain 162: 1126–1134. 33048854 10.1097/j.pain.0000000000002110 PMC7969374

[ptr70303-bib-0068] Ness, T. J. 1999. “Models of Visceral Nociception.” ILAR Journal 40: 119–128. 11406690 10.1093/ilar.40.3.119

[ptr70303-bib-0069] Noor‐Mohammadi, E. , C. O. Ligon , K. Mackenzie , J. Stratton , S. Shnider , and B. Greenwood‐Van Meerveld . 2021. “A Monoclonal Anti‐Calcitonin Gene‐Related Peptide Antibody Decreases Stress‐Induced Colonic Hypersensitivity.” Journal of Pharmacology and Experimental Therapeutics 379: 270–279. 34620725 10.1124/jpet.121.000731

[ptr70303-bib-0070] Ohara, T. E. , and E. Y. Hsiao . 2025. “Microbiota‐Neuroepithelial Signalling Across the Gut‐Brain Axis.” Nature Reviews. Microbiology 23: 371–384. 39743581 10.1038/s41579-024-01136-9

[ptr70303-bib-0071] Palomino‐Pacheco, M. , J. P. Rojas‐Armas , J. M. Ortiz‐Sánchez , J. L. Arroyo‐Acevedo , H. J. Justil‐Guerrero , and J. T. Martínez‐Heredia . 2024. “Assessment of Oral Toxicity of *Moringa oleifera* Lam Aqueous Extract and Its Effect on Gout Induced in a Murine Model.” Veterinary World 17: 1449–1458. 39185060 10.14202/vetworld.2024.1449-1458 PMC11344109

[ptr70303-bib-0072] Pareek, A. , M. Pant , M. M. Gupta , et al. 2023. “ *Moringa oleifera* : An Updated Comprehensive Review of Its Pharmacological Activities, Ethnomedicinal, Phytopharmaceutical Formulation, Clinical, Phytochemical, and Toxicological Aspects.” International Journal of Molecular Sciences 24: 2098. 36768420 10.3390/ijms24032098 PMC9916933

[ptr70303-bib-0073] Pasparakis, M. , and P. Vandenabeele . 2015. “Necroptosis and Its Role in Inflammation.” Nature 517: 311–320. 25592536 10.1038/nature14191

[ptr70303-bib-0074] Perner, C. , and C. L. Sokol . 2021. “Protocol for Dissection and Culture of Murine Dorsal Root Ganglia Neurons to Study Neuropeptide Release.” STAR Protocols 2: 100333. 33615276 10.1016/j.xpro.2021.100333 PMC7876630

[ptr70303-bib-0075] Rajan, T. S. , S. Giacoppo , R. Iori , et al. 2016. “Anti‐Inflammatory and Antioxidant Effects of a Combination of Cannabidiol and Moringin in LPS‐Stimulated Macrophages.” Fitoterapia 112: 104–115. 27215129 10.1016/j.fitote.2016.05.008

[ptr70303-bib-0076] Razis, A. F. A. , R. M. Kamal , G. R. De Nicola , et al. 2025. “Cardio‐ and Neuroprotective Effects by Pretreatment of Dietary Moringin From Moringa Oleifera Seeds and α‐CD/Moringin Formulation in a Rat Model of Isoproterenol‐Induced Myocardial Infarction.” Journal of Nutritional Science 14: e62. 40933258 10.1017/jns.2025.10035 PMC12418292

[ptr70303-bib-0077] Reinecker, H.‐C. , E. Y. Loh , D. J. Ringler , A. Mehta , J. L. Rombeau , and R. P. MacDermott . 1995. “Monocyte‐Chemoattractant Protein 1 Gene Expression in Intestinal Epithelial Cells and Inflammatory Bowel Disease Mucosa.” Gastroenterology 108: 40–50. 7806062 10.1016/0016-5085(95)90006-3

[ptr70303-bib-0078] Sailaja, B. S. , R. Aita , S. Maledatu , D. Ribnicky , M. P. Verzi , and I. Raskin . 2021. “Moringa Isothiocyanate‐1 Regulates Nrf2 and NF‐κB Pathway in Response to LPS‐Driven Sepsis and Inflammation.” PLoS One 16: e0248691. 33793581 10.1371/journal.pone.0248691 PMC8016325

[ptr70303-bib-0079] Saito, Y. , M. Shimizu , K. Iwatsuki , et al. 2021. “Effect of Short‐Time Treatment With TNF‐α on Stem Cell Activity and Barrier Function in Enteroids.” Cytotechnology 73: 669–682. 34349355 10.1007/s10616-021-00487-y PMC8319277

[ptr70303-bib-0080] Schneider, C. A. , W. S. Rasband , and K. W. Eliceiri . 2012. “NIH Image to ImageJ: 25 Years of Image Analysis.” Nature Methods 9: 671–675. 22930834 10.1038/nmeth.2089 PMC5554542

[ptr70303-bib-0081] Schou, W. S. , S. Ashina , F. M. Amin , P. J. Goadsby , and M. Ashina . 2017. “Calcitonin Gene‐Related Peptide and Pain: A Systematic Review.” Journal of Headache and Pain 18: 34. 28303458 10.1186/s10194-017-0741-2 PMC5355411

[ptr70303-bib-0082] Shah, A. , M. Varma , and R. Bhandari . 2024. “Exploring Sulforaphane as Neurotherapeutic: Targeting Nrf2‐Keap & Nf‐Kb Pathway Crosstalk in ASD.” Metabolic Brain Disease 39: 373–385. 37249861 10.1007/s11011-023-01224-4

[ptr70303-bib-0083] Simeoli, R. , G. Mattace Raso , C. Pirozzi , et al. 2017. “An Orally Administered Butyrate‐Releasing Derivative Reduces Neutrophil Recruitment and Inflammation in Dextran Sulphate Sodium‐Induced Murine Colitis.” British Journal of Pharmacology 174: 1484–1496. 27684049 10.1111/bph.13637 PMC5429328

[ptr70303-bib-0084] Singh, S. , D. Anshita , and V. Ravichandiran . 2021. “MCP‐1: Function, Regulation, and Involvement in Disease.” International Immunopharmacology 101: 107598. 34233864 10.1016/j.intimp.2021.107598 PMC8135227

[ptr70303-bib-0085] Singh, S. , R. Bhatia , P. Khare , et al. 2020. “Anti‐Inflammatory Bifidobacterium Strains Prevent Dextran Sodium Sulfate Induced Colitis and Associated Gut Microbial Dysbiosis in Mice.” Scientific Reports 10: 18597. 33122795 10.1038/s41598-020-75702-5 PMC7596498

[ptr70303-bib-0086] Singh, U. P. , N. P. Singh , E. A. Murphy , et al. 2016. “Chemokine and Cytokine Levels in Inflammatory Bowel Disease Patients.” Cytokine 77: 44–49. 26520877 10.1016/j.cyto.2015.10.008 PMC4666758

[ptr70303-bib-0087] Strober, W. , and I. J. Fuss . 2011. “Proinflammatory Cytokines in the Pathogenesis of Inflammatory Bowel Diseases.” Gastroenterology 140: 1756–1767. 21530742 10.1053/j.gastro.2011.02.016 PMC3773507

[ptr70303-bib-0088] Sun, Y. , X. Wang , L. Li , et al. 2024. “The Role of Gut Microbiota in Intestinal Disease: From an Oxidative Stress Perspective.” Frontiers in Microbiology 15: 1328324. 38419631 10.3389/fmicb.2024.1328324 PMC10899708

[ptr70303-bib-0089] Tian, C. , M. Yang , H. Xu , et al. 2023. “Stem Cell‐Derived Intestinal Organoids: A Novel Modality for IBD.” Cell Death Discov 9: 255. 37479716 10.1038/s41420-023-01556-1 PMC10362068

[ptr70303-bib-0090] Trikola, A. 2025. “Patterns and Mechanisms of Visceral and Rectal Hypersensitivity in Patients With Ulcerative Colitis in Remission.” Journal of Crohn's and Colitis 19: i2518.

[ptr70303-bib-0091] van Gils, T. , H. Törnblom , J. P. Hreinsson , B. Jonefjäll , H. Strid , and M. Simrén . 2025. “Factors Associated With Abdominal Pain in Patients With Active and Quiescent Ulcerative Colitis: A Multicohort Study.” Alimentary Pharmacology & Therapeutics 61: 268–277. 39444240 10.1111/apt.18344 PMC11671728

[ptr70303-bib-0092] Vandenabeele, P. , L. Galluzzi , T. Vanden Berghe , and G. Kroemer . 2010. “Molecular Mechanisms of Necroptosis: An Ordered Cellular Explosion.” Nature Reviews. Molecular Cell Biology 11: 700–714. 20823910 10.1038/nrm2970

[ptr70303-bib-0093] Wagner, A. E. , C. Sturm , S. Piegholdt , et al. 2015. “Myrosinase‐Treated Glucoerucin Is a Potent Inducer of the Nrf2 Target Gene Heme Oxygenase 1—Studies in Cultured HT‐29 Cells and Mice.” Journal of Nutritional Biochemistry 26: 661–666. 25776458 10.1016/j.jnutbio.2015.01.004

[ptr70303-bib-0094] Wang, J. , L. Wang , Y. Liu , et al. 2024. “The Keap1‐Nrf2/ARE Signaling Pathway Regulates Redox Balance and Apoptosis in the Small Yellow Croaker ( *Larimichthys polyactis* ) Under Hypoxic Stress.” Science of the Total Environment 957: 177396. 39521089 10.1016/j.scitotenv.2024.177396

[ptr70303-bib-0095] Xia, B. , X. Liu , X. Li , et al. 2022. “Sesamol Ameliorates Dextran Sulfate Sodium‐Induced Depression‐Like and Anxiety‐Like Behaviors in Colitis Mice: The Potential Involvement of the Gut‐Brain Axis.” Food & Function 13: 2865–2883. 35179534 10.1039/d1fo03888e

[ptr70303-bib-0096] Xing, C. , G. Liang , X. Yu , et al. 2023. “Establishment of Epithelial Inflammatory Injury Model Using Intestinal Organoid Cultures.” Stem Cells International 2023: 3328655. 36926182 10.1155/2023/3328655 PMC10014157

[ptr70303-bib-0097] Zhang, S. , Y. Cao , Y. Huang , et al. 2024. “Aqueous *M. oleifera* Leaf Extract Alleviates DSS‐Induced Colitis in Mice Through Suppression of Inflammation.” Journal of Ethnopharmacology 318: 116929. 37480965 10.1016/j.jep.2023.116929

[ptr70303-bib-0098] Zhang, T. , L. Zhao , M. Xu , P. Jiang , and K. Zhang . 2024. “Moringin Alleviates DSS‐Induced Ulcerative Colitis in Mice by Regulating Nrf2/NF‐κB Pathway and PI3K/AKT/mTOR Pathway.” International Immunopharmacology 134: 112241. 38761782 10.1016/j.intimp.2024.112241

