# Supplementary material for: A Combined Colon Organoid‐Sensory Neuron Model Reveals Epithelial Contribution to Moringin Efficacy Against Painful Inflammatory Bowel Disease
Source: Phytother Res. 2026 Mar 23;40(6):3250–66. doi: 10.1002/ptr.70303 (PMC13254238; doi:10.1002/ptr.70303)
Supplement: Supplementary file 1 — Figure S1: Protective effect of bioactivated glucomoringin (GMG + MYR) on DSS‐induced colonic damage. Representative images of H&E‐stained sections (×40 magnification; scale bar: 500 μm) are shown to allow the visualization of the entire colonic section and ensure a representative overview of tissue damage. For each image, the respective inserts (×100 magnification; scale bar: 200 μm) highlight inflammatory cell infiltration, crypt architecture, goblet cell depletion, and epithelial integrity. [file PTR-40-3250-s001.docx]

**A combined colon organoid-sensory neuron model reveals epithelial contribution to moringin efficacy against painful inflammatory bowel disease**

Francesco Margiotta ^a, b, #^, Elena Lucarini ^a, #,^ *, Alessandra Toti ^a^, Maria Giovanna Cataldi ^a^, Clara Ciampi ^a^, Gina Rosalinda De Nicola ^c^, Lorenzo Di Cesare Mannelli ^a^, Carla Ghelardini ^a^

^a^ Department of Neuroscience, Psychology, Drug Research and Child Health - NEUROFARBA - Pharmacology and Toxicology Section, University of Florence, Viale Gaetano Pieraccini 6, 50139, Florence, Italy.

^b^ European Biomedical Research Institute of Salerno (EBRIS), Via Salvatore de Renzi 50, 84125 Salerno, Italy.

^c^ Research Centre for Vegetable and Ornamental Crops, Council for Agricultural Research and Economics (CREA), Via dei Fiori 8, Pescia, 51017, Italy

^#^ These authors contributed equally to this work

* Correspondence to: Dr. Elena Lucarini, Department of Neuroscience, Psychology, Drug Research and Child Health - NEUROFARBA - Pharmacology and Toxicology Section, University of Florence, Viale Gaetano Pieraccini 6, 50139, Florence, Italy; Phone: +39 0552758395; E-mail: [elena.lucarini@unifi.it](mailto:elena.lucarini@unifi.it)

Short title: Colon organoid system predicts moringin efficacy in IBD
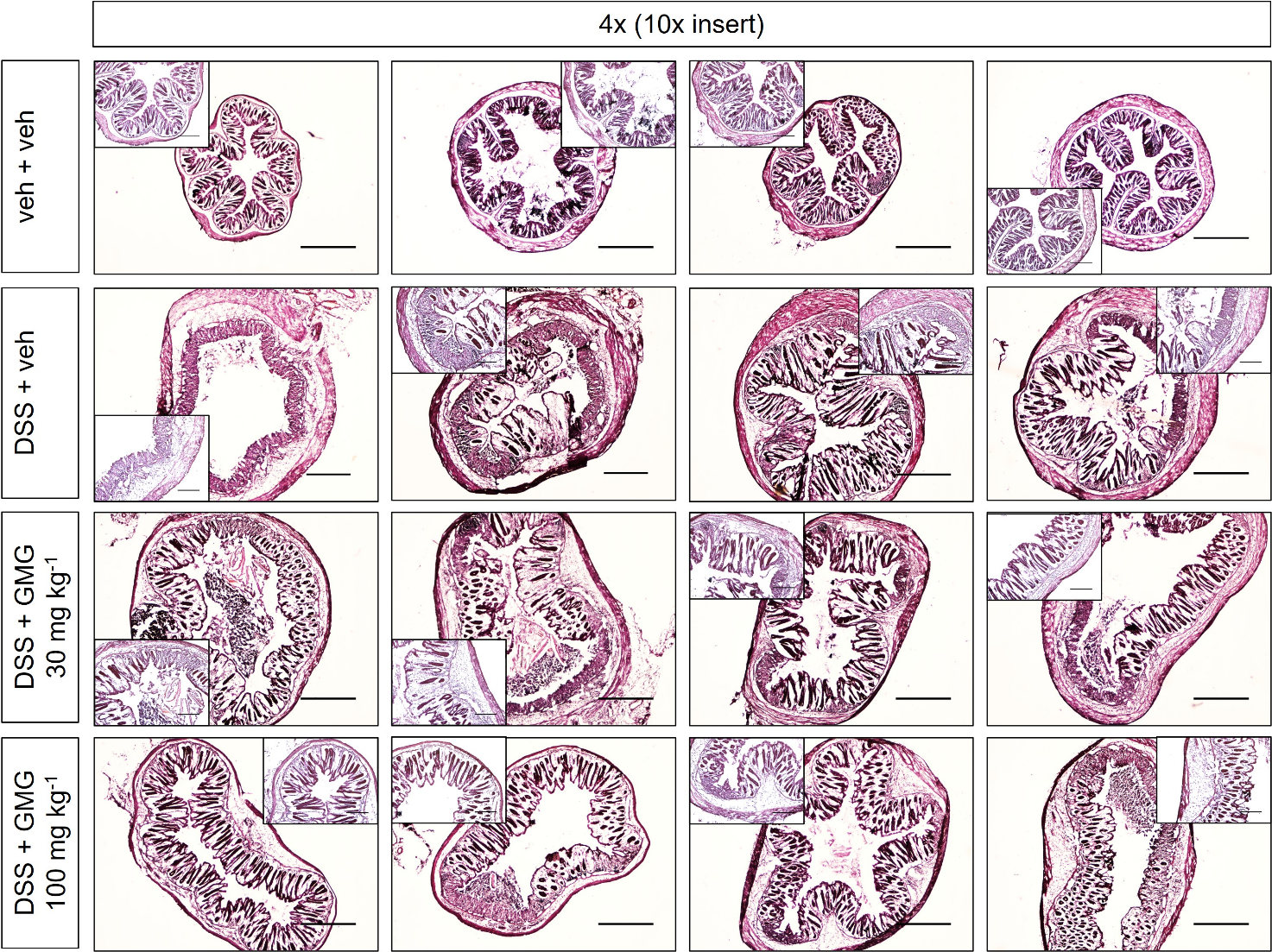


**Figure S1. Protective effect of bioactivated glucomoringin (GMG + MYR) on DSS-induced colonic damage.** Representative images of H&E-stained sections (40x magnification; scale bar: 500 µm) are shown to allow the visualization of the entire colonic section and ensure a representative overview of tissue damage. For each image, the respective inserts (100x magnification; scale bar: 200 µm) highlight inflammatory cell infiltration, crypt architecture, goblet cell depletion, and epithelial integrity.
